# Zfp281 Functions as a Transcriptional Repressor for Pluripotency of Mouse Embryonic Stem Cells

**DOI:** 10.1002/stem.736

**Published:** 2011-09-13

**Authors:** Miguel Fidalgo, P Chandra Shekar, Yen-Sin Ang, Yuko Fujiwara, Stuart H Orkin, Jianlong Wang

**Affiliations:** a Department of Developmental and Regenerative Biology, Black Family Stem Cell Institute, Mount Sinai School of MedicineNew York, USA; b Children's Hospital and Dana Farber Cancer Institute, Harvard Medical SchoolBoston, Massachusetts, USA; c Harvard Stem Cell InstituteCambridge, Massachusetts, USA; d Howard Hughes Medical InstituteBoston, Massachusetts, USA

**Keywords:** Zfp281, Nanog, Embryonic stem cells, Transcriptional repressor, Self-renewal, Pluripotency

## Abstract

Embryonic stem cells (ESCs) derived from preimplantation blastocysts have unique self-renewal and multilineage differentiation properties that are controlled by key components of a core regulatory network including Oct4, Sox2, and Nanog. Understanding molecular underpinnings of these properties requires identification and characterization of additional factors that act in conjunction with these key factors in ESCs. We have previously identified Zfp281, a Krüppel-like zinc finger transcription factor, as an interaction partner of Nanog. We now present detailed functional analyses of Zfp281 using a genetically ablated null allele in mouse ESCs. Our data show that while Zfp281 is dispensable for establishment and maintenance of ESCs, it is required for their proper differentiation in vitro. We performed microarray profiling in combination with previously published datasets of Zfp281 global target gene occupancy and found that Zfp281 mainly functions as a repressor to restrict expression of many stem cell pluripotency genes. In particular, we demonstrated that deletion of Zfp281 resulted in upregulation of Nanog at both the transcript and protein levels with concomitant compromised differentiation of ESCs during embryoid body culture. Chromatin immunoprecipitation experiments demonstrated that Zfp281 is required for Nanog binding to its own promoter, suggesting that Nanog-associated repressive complex(es) involving Zfp281 may fine-tune Nanog expression for pluripotency of ESCs.

## INTRODUCTION

Embryonic stem cells (ESCs) are pluripotent cells derived from the inner cell mass of blastocysts [1]. ESCs can self-renew and retain their potential to differentiate into all cell types of the three germ layers, a phenomenon often referred to as pluripotency. Self-renewal and pluripotency of ESCs are regulated by the core transcription factors Oct4, Nanog, and Sox2 [2] along with other genetic and epigenetic factors [3]. Together they form a transcriptional regulatory network controlling the expression of many downstream target genes to establish and maintain pluripotency of ESCs [4–6]. The core transcription factors, particularly Nanog and Oct4, physically interact with each other and with other transcription factors [7–9] linking multiple corepressor complexes [10–12]. Loss of function for many of these interacting proteins leads to loss of pluripotency and/or early embryonic defects [7–9, 11] underscoring functional significance of additional factors in regulation of pluripotency. Therefore, dissecting the functions of various interacting partners of the core pluripotency factors will unravel the intricate details of the mechanisms governing ESC pluripotency.

The Krüppel-like zinc finger transcription factor Zfp281 was identified as an important interacting partner of Nanog in our previous study [9] and was subsequently confirmed also to be a partner of Oct4 and Sox2 [13]. The human homolog ZNF281 (also known as ZBP-99) was first cloned and identified as a transcriptional repressor binding to GC-rich promoters in human cells [14]. Studies so far have suggested that it mainly functions as a transcriptional repressor for regulation of downstream target genes [15, 16]. Zfp281/ZNF281 belongs to a novel class of transcription factors that contain a characteristic array of four Krüppel type zinc fingers and are phylogenetically conserved in mammals [14]. It has been shown that ZNF281 RNA is expressed ubiquitously at low levels, with elevated expression levels in placenta and in adult kidney, liver, and lymphocytes [15].

Several studies have implicated Zfp281/ZNF281 in regulating stem cell pluripotency and developmental processes. First, an earlier study [17] using transcriptomic profiling of human ESCs and their differentiated progenies identified ZNF281 as one of the upregulated transcriptional regulators in undifferentiated human ESCs. Second, a study of c-Myc-associated proteins in both colorectal cancer cells and human embryonic kidney cells identified ZNF281 as a novel c-Myc interacting partner [18]. c-Myc is an oncogene encoding a transcription factor that is important for stem cell maintenance [19] and induced pluripotency [20]. Third, Zfp281 has been identified and validated as a top candidate target of the skin microRNA-203, which promotes differentiation and represses stemness in epidermis [21]. This suggests that Zfp281 may also play a critical role in adult stem cell maintenance and in skin development. Fourth, genome-wide promoter analysis using chromatin immunoprecipitation (ChIP) combined with chip technology (ChIP-chip) of the SOX4 transcriptional network in human prostate cancer cells has identified ZNF281 as one of the direct transcriptional targets of SOX4, a critical developmental transcription factor required for precise differentiation and proliferation in multiple tissues and dysregulated in many types of human cancers [22]. Taken together, Zfp281/ZNF281 likely plays diverse roles in cell proliferation and differentiation, oncogenesis, stem cell maintenance, embryogenesis, and development.

The expression of Zfp281 is enriched in mouse ESCs relative to differentiated cells [13] and its promoter is bound by Oct4, Nanog, and Sox2 [23, 24], which suggests a possible function in maintenance of ESC pluripotency. Zfp281 binds to many common target genes bound by Oct4, Nanog, and Sox2 supporting its important role in transcriptional regulation of pluripotency [5, 13]. To further understand Zfp281 function in stem cell pluripotency, we report in this study the generation of a targeted Zfp281 allele in mouse ESCs as well as derivation and characterization of Zfp281 deficient ESCs. We found that, while Zfp281 is essential for early postimplantation mouse embryogenesis, Zfp281 is dispensable for establishment and maintenance of ESCs but required for their proper differentiation. We performed microarray analyses combined with available Zfp281 target gene occupancy datasets and demonstrated that Zfp281 functions as a transcriptional repressor to restrict expression of many pluripotency genes including *Nanog* in ESCs, consistent with our previous RNAi study [9]. We also found that depletion of Zfp281 results in delayed downregulation of stem cell markers and compromised differentiation during embryoid body (EB) culture. Finally, we performed ChIP to demonstrate that Zfp281 is required for Nanog binding to its own promoter and proposed a model to explain the repressor function of Zfp281 in Nanog transcriptional regulation of stem cell pluripotency.

## MATERIALS AND METHODS

### Generation of a *Zfp281* Gene Targeted Allele and Derivation of *Zfp281* Knockout ESC Lines

**Figure 1:**
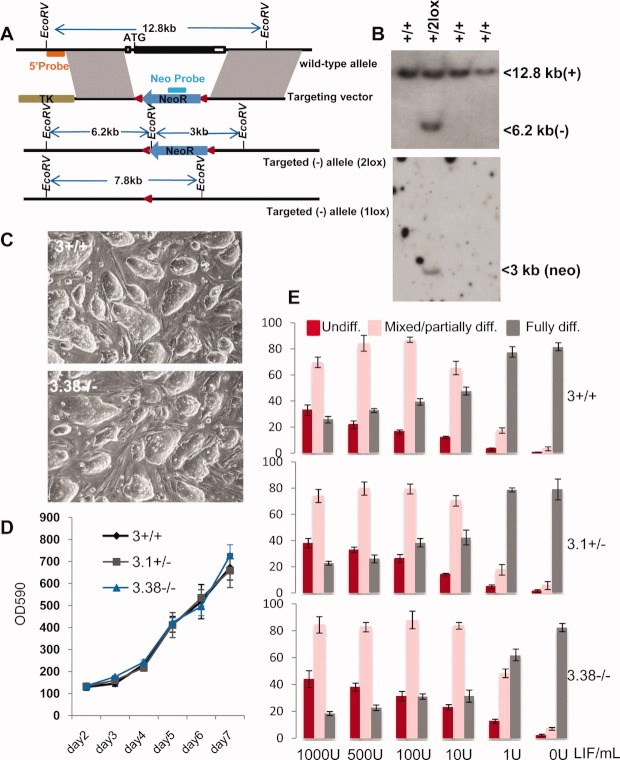
Establishment and characterization of Zfp281 deficient embryonic stem cell (ESC) lines. (A): Schematic depiction of gene targeting strategy for generation of the null allele of Zfp281. A targeting vector carrying a floxed neomycin resistance gene (neoR) cassette flanked by homologous sequences outside the two exons of Zfp281 was used to replace the Zfp281 gene in ESCs. Upon targeting, both exons of Zfp281 were replaced by a floxed NeoR cassette deleting all the coding regions of the gene. The orange and blue bars denote external and internal probes, respectively, for Southern hybridization. (B): Southern blot analyses of the ESC clones obtained from the Zfp281 targeting experiment. A probe external to the 5′ sequence of the targeting vector hybridizes to a 12.8-kb EcoRV fragment in the wild-type allele. Upon targeting, it hybridizes to a 6.2-kb fragment (2lox). The neoR cassette can be removed by transient Cre recombinase expression to generate the 7.8 kb null (1lox) allele (Supporting Information Fig. S1A). The same membrane was stripped off and rehybridized with a neo probe to confirm the correct targeting. (C): Morphology of Zfp281 deficient ESCs (bottom) compared with wild-type ESCs (top). (D): Growth curve 3-(4,5-dimethylthiazol-2-yl)-2,5-diphenyltetrazolium bromide (MTT) analysis of the wild-type, heterozygous and null ESCs. Error bars represent SD. (E): Colony formation assay of wild-type, heterozygous, and null ESCs. Cells were cultured at clonal density in the presence of a series of leukemia inhibitory factor (LIF) concentrations as indicated for 6 days and colonies were scored as fully undifferentiated (red), mixed or partially differentiated (pink), and fully differentiated (gray) based on the extent of alkaline phosphatase staining. Error bars represent SD.

To construct the targeting vector, we amplified homologous arms from CJ7 ESC genomic DNA by polymerase chain reaction (PCR) for the left and right homologous arms, respectively. The PCR products were sequence verified and cloned into the pLNTK vector (a gift from the Alt Lab in Children's Hospital Boston). The final targeting vector was linearized with a *Pvu*I restriction site and electroporated into CJ7 ESCs. Drug selection (300 μg/ml G418) was applied 24 hours after electroporation and drug resistant clones were picked after 10 days. Positive clones were identified by Southern blotting with the two probes that are external and internal, respectively, to the targeting vector (Fig. 1A). Targeted clones with the normal karyotype were injected into host C57BL/6 blastocysts to make chimeric mice for germline transmission. Mice heterozygous for the targeted Zfp281 allele were identified by Southern blotting. Embryo manipulations and blastocyst outgrowth assays were performed as described [25]. The genotypes of derived lines were confirmed by Southern hybridization.

### ESC Culture, Colony Formation, and MTT Assays

All ESC lines were cultured as described [9] and colony formation assay was performed as described [26]. ESC proliferation assay was done by plating approximately 200 cells in 96 wells in 100 μl ESC medium. 3-(4,5-Dimethylthiazol-2-Yl)-2,5-diphenyltetrazolium bromide (MTT) of 5 mg/ml (20 μl) was added to each well and the plate was incubated at 37°C for 2 hours. The media was removed and the incorporated formazan crystals were dissolved in 200 μl dimethyl sulfoxide (DMSO). The absorbance of the dissolved dye was read at 590 nm. The proliferation curve was obtained by plotting the absorbance over a time course of 7 days.

### EB Differentiation and Analysis

ESCs were adapted to feeder free culture conditions for five passages to completely deplete any remaining feeder cells. Approximately 1 × 10^6^ ESCs were cultured in suspension in low attachment bacterial grade petri dishes containing ESC medium without leukemia inhibitory factor (LIF). The medium was replenished every other day and EBs were harvested for RNA analysis. For size comparison of individual EBs, 11 randomly selected day 6 EBs each from wild-type and null ESCs were scored, imaged, and measured directly on screen (zoomed 50%) using Microsoft Office Document Imaging software.

### Cell Cycle and Apoptosis Analysis

Cell cycle analysis was performed by staining the DNA content of ESCs with propidium iodide (PI). ESCs were resuspended to single cells by trypsin treatment and then fixed in ice cold 70% ethanol for 1 hour at 4°C. Cells were treated with 50 μg/ml PI and 0.1 mg/ml RNase A for 40 minutes at 37°C. Cells were then washed with phosphate buffer saline (PBS) and the cell cycle profile was acquired by flow-cytometry using an LSR-II (BD Bioscience, San Jose, CA, www.bdbiosciences.com).

For apoptosis analysis, ESCs were resuspended to single cells by trypsin treatment and stained with PI and Annexin V-enhanced green fluorescent protein (EGFP) (GenScript, Piscataway, NJ, www.genscript.com) for 10 minutes. Cells were then washed with PBS and the apoptotic profile was acquired by flow-cytometry. Fluorescence-activated cell sorting (FACS) analysis data was analyzed using FlowJo software.

### Immunostaining and Western Blot Analysis

Cells were grown on cover slips and fixed for 10 minutes in 4% paraformaldehyde followed by permeabilization with 0.5% Triton X-100 for 6 minutes at room temperature. Cells were blocked with 3% horse serum in PBS and then incubated with a primary anti-Nanog antibody (Cosmo Bio USA, Inc, Carlsbad, CA, http://www.cosmobiousa.com) for 1 hour at room temperature. For Western blotting analysis, total protein was extracted with radioimmunoprecipitation assay buffer (RIPA) buffer containing 1x protease inhibitor cocktail (Roche Applied Science, Indianapolis, IN, https://www.roche-applied-science.com) and resolved on a 4%–20% gradient polyacrylamide gel. Protein was transferred onto a polyvinylidene fluoride (PVDF) membrane and hybridized with anti-Nanog (Chemicon, Millipore, Billerica, MA, www.millipore.com), anti-Oct4 (Santa Cruz Biotechnology, Santa Cruz, CA, www.scbt.com), and anti-β-actin (Abcam, Cambridge, MA, www.abcam.com/) antibodies.

### ChIP and qPCR

ChIP was performed on wild-type, heterozygous, and null ESCs using an anti-Nanog antibody (Cosmo Bio USA) as described previously [27]. Quantitative PCR (qPCR) analysis was done using the LightCycler 480 SYBR Green I Master System (Roche). Relative enrichment of regions of interest was calculated in comparison with the enrichment of unrelated regions (glyceraldehyde 3-phosphate dehydrogenase (GAPDH) and β2-microglobulin). All primers used in this study are listed in Supporting Information Table 1.

### Gene Expression, Microarray, and GeneSet Enrichment Analyses

RNA was isolated from ESCs (and EBs) using Trizol (Invitrogen, Carlsbad, CA, www.invitrogen.com), according to the manufacturer's instructions. Reverse transcription was performed with the Superscript III First-Strand Synthesis System (Invitrogen). qPCR for marker gene expression analysis was performed as described above.

Microarray analysis was performed using Affymetrix mouse genome 430 2.0 array chips. All arrays were robust multi-array analysis (RMA) normalized. Differentially expressed genes (Log_2_ fold change >1 for upregulated, <−1 for downregulated) were identified, using Limma. Heat maps were generated by hierarchical clustering using Cluster 3.0 software and visualized using TreeView software. The microarray data have been deposited to the public domain (gene expression omnibus (GEO) accession# GSE30293).

Gene ontology (GO) analysis was performed using the DAVID online bioinformatics tool (http://david.abcc.ncifcrf.gov/). Gene set enrichment analysis (GSEA) was performed with GSEA software (http://www.broadinstitute.org/gsea/) using the following parameters: permutation, phenotype; metric, Signal2Noise; metric, weighted; and #permutation, 1,000. Venn diagrams are not plotted to scale.

## RESULTS

### Derivation and Characterization of Zfp281 Null ESCs

To introduce a null mutation at the *Zfp281* locus, we replaced the entire *Zfp281* gene comprising two exons with a floxed neomycin resistance gene and identified clones with correctly targeted alleles by Southern hybridization (Fig. 1A, 1B). Heterozygous ESCs with normal karyotype were injected into wild-type blastocysts to generate chimeras for germline transmission of the mutant allele. The resulting heterozygous mice were phenotypically normal, and staged embryo analysis of the heterozygous matings indicated that Zfp281 null embryos die between embryonic day 7.5 (E7.5) and E8.5 (data not shown), suggesting that mutant Zfp281 ESC lines may be derived by outgrowth of the E3.5 blastocysts from heterozygous matings. Indeed, we successfully derived multiple ESC lines with wild-type, heterozygous, and homozygous *Zfp281* alleles from three independent experiments (Fig. 1; Supporting Information Fig. S1 and Table S1). The null status of these mutant ESCs was further confirmed by the absence of Zfp281 transcripts in a reverse transcriptase (RT)-PCR assay (Supporting Information Fig. S1B).

To address if loss of Zfp281 affects ESC self-renewal, we performed alkaline phosphatase (AP) staining, apoptosis, cell cycle profile, and growth curve analyses in wild-type (+/+), heterozygous (+/−), and null (−/−) ESCs. We found that, similar to wild-type ESCs, Zfp281 null ESCs maintain characteristic ESC morphology (Fig. 1A) and are stained positive for AP activity under standard culture conditions (Supporting Information Fig. S1E), indicating mutant ESCs maintain an undifferentiated self-renewal state. We also found that the percentage of apoptotic cells in null ESCs was similar to that of wild-type and heterozygous ESCs (Supporting Information Fig. S1D), suggesting that the loss of Zfp281 does not affect ESC survival. Cell cycle profile analysis showed no significant differences in the cell cycle distribution of wild-type, heterozygous, and null ESCs (Supporting Information Fig. S1C). Furthermore, the proliferation of ESCs was analyzed over a period of 7 days by an MTT assay. Again, we did not observe a significant difference in the proliferation rates of multiple null ESCs relative to wild-type and heterozygous ESCs (Fig. 1A; Supporting Information Fig. S1F). These data suggest that Zfp281 is dispensable for survival and proliferation of ESCs.

To measure self-renewal of ESCs at the single cell level, we cultured cells in the presence and absence of LIF at clonal density and scored the colonies as undifferentiated, partially differentiated (mixed), and differentiated according to AP staining patterns. In the presence of 1,000 U/ml LIF, we observed statistically more undifferentiated and partially differentiated or mixed colonies and less fully differentiated colonies formed in null ESCs (3.34−/−) than in the wild-type (3.3+/+) and heterozygous (5+/−) ESCs (Supporting Information Fig. S1G, left). This result suggests that self-renewal of Zfp281 null ESCs was maintained and even slightly enhanced under standard ESC culture conditions. We also performed the colony formation assay in the absence of LIF, and observed no significant difference in colony formation among all three cell lines tested (Supporting Information Fig. S1G, right), indicating that the withdrawal of LIF did not have an additional impact on the self-renewal of null ESCs relative to their wild-type and heterozygous counterparts. We further tested the mutant ESCs for their sensitivity to the LIF concentration in the colony formation assay using independently derived lines. Under 10 and 1 U/ml of LIF, we detected relatively more undifferentiated and partially differentiated colonies and less fully differentiated colonies in mutant ESCs (3.38−/−) compared with wild-type (3+/+) and heterozygous (3.1+/−) ESCs (Fig. 1A), indicating a slight enhancement of self-renewal of the Zfp281 null ESCs. Together, our results demonstrate that Zfp281 is dispensable for derivation and maintenance of ESCs and may negatively regulate the self-renewal state of ESCs.

### Dysregulation of Pluripotency and Lineage Specific Markers in Zfp281 Null ESCs

**Figure 2:**
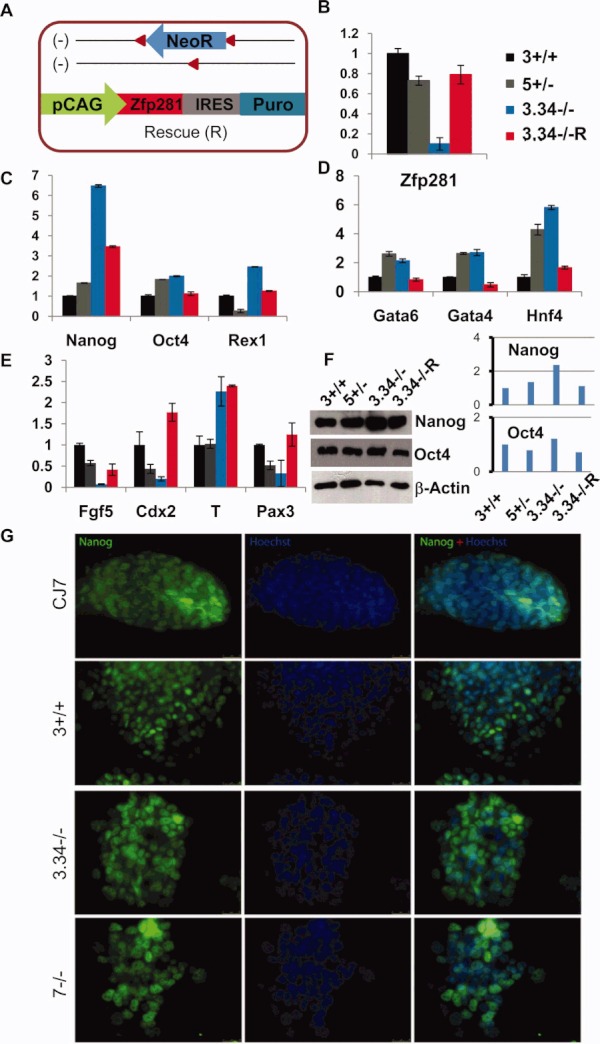
Dysregulation of pluripotency and lineage specific markers in Zfp281 deficient embryonic stem cells (ESCs). (A): Depiction of the strategy for transgenic rescue of Zfp281 deficient ESCs with the pPyCAG-Zfp281IP expression vector. The two null (−) alleles (with and without the neomycin resistance gene) are shown. (B): Quantitative reverse transcriptase-polymerase chain reaction (RT-PCR) analyses of expression levels of Zfp281 transcripts in wild-type, heterozygous, null, and rescued ESCs. Note that the rescue level of Zfp281 is approximately 80% of the wild-type level. “3.34−/−R” represents Zfp281 deficient ESCs in which expression of Zfp281 is restored by ectopic expression of transgenic Zfp281 cDNA. Error bars represent SD. (C): Quantitative RT-PCR for relative expression levels of pluripotency markers in ESCs. (D): Quantitative RT-PCR for relative expression levels of endodermal markers in ESCs. (E): Quantitative RT-PCR for relative expression levels of differentiation markers in ESCs. (F): Western blot analysis showing a higher level of Nanog but a relatively smaller increase of Oct4 expression in Zfp281 deficient ESCs. β-Actin was used as a loading control. Western data were scanned and density of target bands was quantified using the ImageJ Software of the NIH. Band density was normalized to that of the β-actin loading control. (G): Heterogeneous expression of Nanog in wild-type, heterozygous, and null ESCs. Note that expression of Nanog in Zfp281 null ESCs is still as heterogeneous as that in wild-type controls (CJ7 and 3+/+).

To address how loss of Zfp281 affects expression of pluripotency and lineage specific genes, we analyzed the expression levels of various markers in the derived ESC lines. To ensure that dysregulation of marker gene expression is primarily due to loss of Zfp281, we rescued one of the null ESC lines with ectopic expression of Zfp281 cDNA under the control of the constitutively active CAG promoter (Fig. 2A). We identified a rescue clone that restored approximately 80% of wild-type level of *Zfp281* (Fig. 2A). The expression levels of pluripotency genes were upregulated by sixfold for *Nanog* and twofold each for *Oct4* and *Rex1* in the Zfp281 null ESCs (Fig. 2A). The introduction of transgenic Zfp281 restored their expression to levels closer or equivalent to wild-type levels (Fig. 2A). Interestingly, despite a higher expression level of *Nanog* in null ESCs, the expression of endoderm markers *Gata6*, *Gata4*, and *Hnf4* was also derepressed in both heterozygous and null ESCs and were restored to wild-type levels upon Zfp281 transgene expression (Fig. 2A), suggesting that the dysregulation of stem cell and endoderm markers observed in the null ESCs is the direct consequence of Zfp281 loss. The dysregulation of marker gene expression was also observed for other lineages such as primitive ectoderm (*Fgf5*), mesoderm (*T*), ectoderm (*Pax3*), and trophectoderm (*Cdx2*) (Fig. 2A). Ectopic expression of Zfp281 cDNA restores the expression levels of some but not all markers to wild-type levels, suggesting that dysregulation of some lineage specific markers could be due to a secondary effect upon Zfp281 loss. Alternatively, the 80% of wild-type level transgenic expression might be insufficient to achieve a complete rescue.

The slightly enhanced self-renewal of Zfp281 null ESCs (Fig. 1; Supporting Information Fig. S1) and pronounced upregulation of *Nanog* RNA expression in these cells (Fig. 2A) prompted us to further examine its expression at the protein level. We confirmed the upregulation of Nanog (approximately twofold) but a minimal increase of Oct4 expression by Western blot analysis in the mutant line (Fig. 2A). We performed similar studies in other independently derived lines and found that the data (Supporting Information Fig. S2A–S2E) are largely consistent with the above findings with minimal clonal variations for certain markers such as Rex1 and T in heterozygous and null cells, respectively. Therefore, our results demonstrate that Zfp281 is required for proper control of pluripotency and lineage marker gene expression in ESCs. Loss of Zfp281 leads to upregulation of several stem cell markers, particularly *Nanog*, and dysregulation of multiple lineage specific marker gene expression, particularly, derepression of the endoderm marker gene expression.

Nanog is heterogeneously expressed in ESCs, and the expression level of Nanog in individual ESCs dictates their self-renewal versus differentiation propensity [28]. To address whether upregulation of Nanog in Zfp281 null ESCs affects its heterogeneous expression pattern, we performed Nanog immunostaining in these ESCs (Fig. 2A). We confirmed the heterogeneous expression pattern of Nanog in wild-type ESCs (CJ7 and 3+/+) (top two panels), and importantly, found that Zfp281 depletion in two independent null ESCs (3.34−/− and 7−/−) does not alter the heterogeneous expression pattern of Nanog (Fig. 2A, bottom two panels). We also confirmed this expression pattern of Nanog in Zfp281 heterozygous and rescued mutant ESCs (Supporting Information Fig. S2F). These data suggest that Zfp281 likely fine-tunes Nanog expression in preexisting Nanog-expressing cells and prevents its excessive expression to maintain pluripotency of ESCs.

### Zfp281 Functions As a Transcriptional Repressor for Pluripotency Factors in ESCs

**Figure 3:**
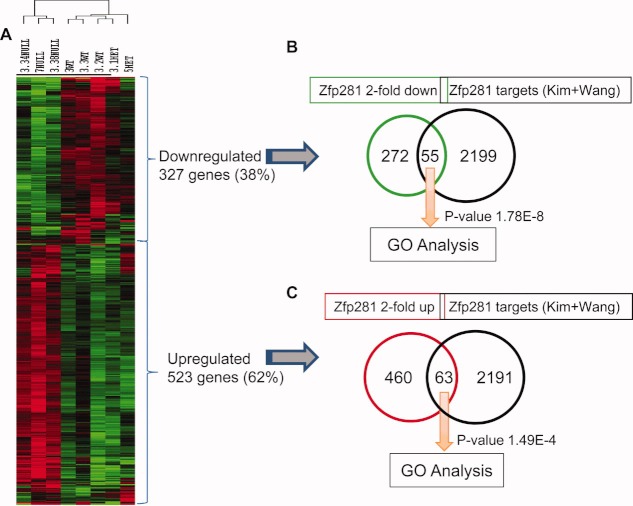
Transcriptional profiling and target gene regulation upon Zfp281 depletion. (A): A heatmap depiction of expression levels of all the genes with at least a twofold alteration (downregulation or upregulation) between wild-type and null embryonic stem cells. (B): Intersection of downregulated genes with direct target genes of Zfp281 identified by Kim et al. [5] and Wang et al. [13]. (C): Intersection of upregulated genes with direct target genes of Zfp281 identified by Kim et al. [5] and Wang et al. [13]. Abbreviation: GO, gene ontology.

To gain an overview of the global effect of Zfp281 depletion on the ESC transcriptome, we performed microarray analysis using multiple lines of wild-type, heterozygous and null ESCs. With a twofold cutoff for changes in gene expression, we found that a total of 850 genes are differentially expressed in Zfp281 null ESCs. Among them, 327 (38%) genes were downregulated and 523 (62%) genes were upregulated upon Zfp281 depletion (Fig. 3A). To examine how many differentially expressed genes are direct transcriptional targets of Zfp281, we compared our microarray data with two previously published ChIP-chip datasets [5, 13] for Zfp281 target genes. We found that among 2,254 potential Zfp281 target genes from the two studies, 55 were positively regulated by Zfp281, that is, downregulated upon Zfp281 depletion (Fig. 3A), whereas 63 were negatively regulated, that is, upregulated upon Zfp281 depletion (Fig. 3A). These results suggest that Zfp281 can act both as a transcriptional activator and a repressor, a conclusion consistent with a prior study [13]. To gain molecular insights into the function of Zfp281 in transcriptional regulation, we performed GO analysis of these differentially regulated, direct target genes. We found that the downregulated genes are largely involved in molecular functions such as transcription regulator activity and participate in biological processes such as development as well as other cellular processes including cell signaling and adhesion (Supporting Information Fig. S3A). In contrast, the upregulated genes are largely involved in molecular functions such as RNA/chromatin binding, transcription factor/cofactor binding, and repressor/corepressor activity. Importantly, these genes appear to predominantly participate in biological processes with a negative regulator function (Supporting Information Fig. S3B). These results are consistent with transcription factor activity and the presumed repressor function of Zfp281.

**Figure 4:**
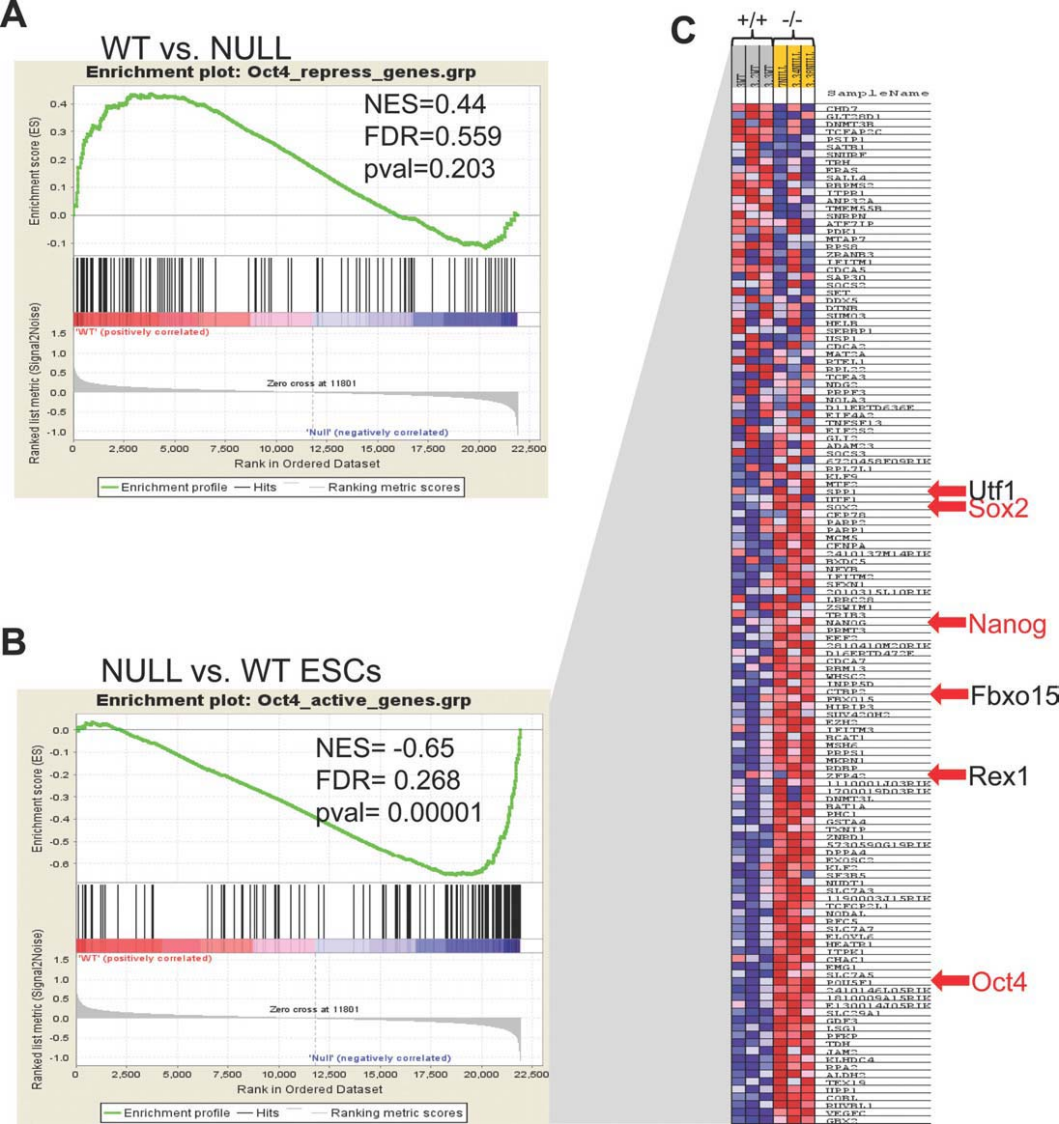
Gene set enrichment analysis (GSEA) of Zfp281 wild-type versus null embryonic stem cells (ESCs). (A,B): GSEA analyses of gene sets representing Oct4-repressed (A) and Oct4-activated (B) genes. Oct4-repressed genes are enriched in wild-type but become repressed upon Zfp281 depletion (NULL); Oct4-activated genes are significantly enriched in Zfp281 null ESCs and are further upregulated upon Zfp281 depletion. (C): Heatmap showing enriched genes in the Oct4-activated GSEA. A complete list of genes is provided in Supporting Information Table 3. Representative key genes are highlighted. Abbreviations: WT, wild-type; ESC, embryonic stem cell; NES, normalized enrichment score; pval, nominal *p*-value; FDR, false discovery rate.

To further extract biological insight from our microarray dataset, we performed GSEA of ranked differentially expressed gene lists on two predefined subsets of Oct4 target genes (Fig. 4). The set of “Oct4_repress_genes” are those Oct4-bound targets [29] whose expression levels are upregulated upon Oct4 knockdown [30], which consist of many developmental regulators and lineage specific transcription factors. The set of “Oct4_active_genes” are those Oct4-bound target genes that are downregulated upon Oct4 knockdown, which consist of many self-renewal regulators and pluripotency factors. We found that the “Oct4_repress_genes” set is relatively enriched in wild-type ESCs, but is further repressed upon Zfp281 depletion, albeit with weak statistical significance (Fig. 4A). In contrast, the “Oct4_active_genes” set is significantly more enriched in Zfp281 null ESCs (Fig. 4A), suggesting that Zfp281 functions mainly as a transcriptional repressor in ESCs. Consistent with this, we found that many self-renewal regulators and pluripotency factors such as Nanog, Oct4, and Sox2 as well as the ESC markers Utf1, Rex1, and Fbxo15 are upregulated in null ESCs (Fig. 4A). The differential expression of a selected number of both downregulated and upregulated genes was further confirmed by qPCR using two independently derived pairs of wild-type/null ESC lines (Supporting Information Fig. S4A–S4E). In particular, we confirmed upregulation of the stem cell active genes *Nanog*, *Oct4*, and *Tbx3*, downregulation of the trophectoderm markers *Cdx2, Rhox6*, and *Elf5*, and derepression of endoderm markers *Gata6*, *Cxcr4*, and *Sox17* in Zfp281 null ESCs, consistent with our qPCR analyses (Fig. 2; Supporting Information Fig. S2). Together our results unambiguously argue for a transcriptional repressor function of Zfp281 in regulating self-renewal and pluripotency of ESCs.

### Zfp281 Deficient ESCs Show Abnormal EB Differentiation

**Figure 5:**
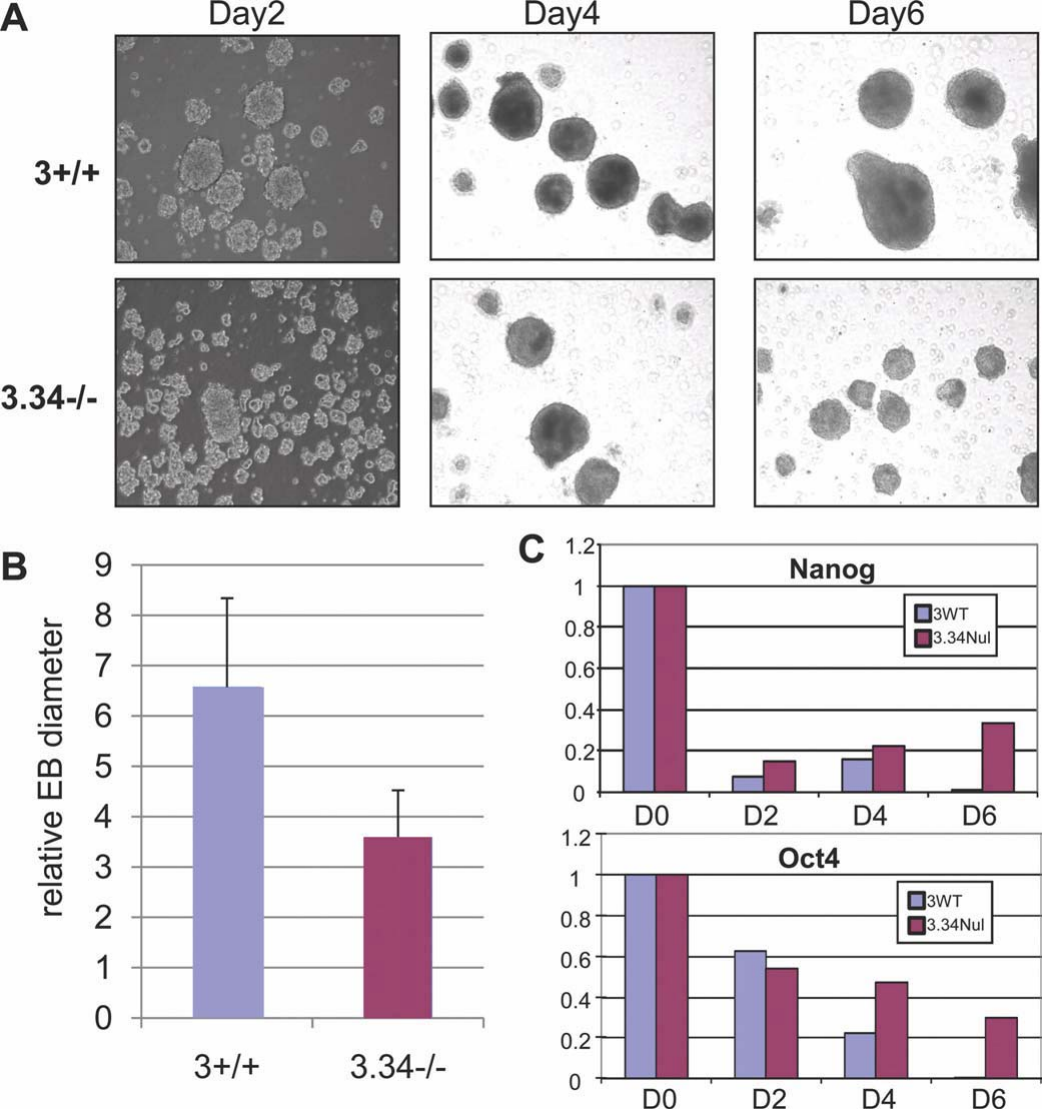
Abnormal in vitro differentiation of Zfp281 deficient embryonic stem cells (ESCs). (A): Morphology of embryoid bodies (EBs) during the time course of differentiation of wild-type and null ESCs. (B): Analysis of the sizes of wild-type (3+/+) and Zfp281 null (3.34−/−) EBs at day 6. Eleven each of randomly selected wild-type and null EBs were scored and their relative diameters from images taken under identical magnification were calculated. The data were presented with the average sizes of the wild-type and null EBs, and the error bars denote SD. (C): Quantitative reverse transcriptase-polymerase chain reaction for relative expression levels of pluripotency genes (*Nanog* and *Oct4*) during the time course of EB differentiation. For both genes, their expression levels in ESCs (at day 0) were arbitrarily set one and their relative expression levels during EB differentiation were normalized to the mRNA levels at day 0. Note that the actual overall expression levels of both genes are higher in 3.34 Null ESCs (red bars) than in three WT ESCs (blue bars) (Supporting Information Fig. S5B). Abbreviations: EB, embryoid body; WT, wild-type.

The above microarray and GSEA data are consistent with our initial observation that Zfp281 deficient ESCs exhibit a certain degree of enhanced self-renewal (Fig. 1; Supporting Information Fig. S1). The aberrant marker gene expression pattern (Fig. 2; Supporting Information Fig. S2) and upregulation of stem cell active genes in Zfp281 null ESCs (Fig. 4; Supporting Information Fig. S4) prompted us to examine the differentiation properties of these cells. We differentiated ESCs into EBs and analyzed EB morphology by microscopy. Our results show that null ESCs differentiate into EBs, however, these EBs are smaller and less well developed than their wild-type counterparts (Fig. 5A). We randomly chose 11 EBs of 6-day culture and scored their relative diameters microscopically. Our results indicate that null ESCs formed EBs with an average size half that of the wild-type counterparts (Fig. 5A). Similar morphological differences were observed with another null ESC line that was monitored over a 12-day period (Supporting Information Fig. S5A, left panels), although the EB size differences between wild-type and null samples were reduced during late differentiation stages (Supporting Information Fig. S5A, right panel). In addition, the cysts in the null EBs were observed only at day 12 in contrast to day 10 in the wild-type EBs (Supporting Information Fig. S5A; data not shown). These morphological defects in the null EBs suggested a delayed differentiation of Zfp281 deficient ESCs.

Proper downregulation of self-renewal regulators such as Oct4 and Nanog is necessary for ESCs to differentiate into multiple lineages. We asked if the delayed differentiation of Zfp281 null ESCs might be due to insufficient downregulation of Nanog and Oct4 in these cells. Indeed, qPCR analyses of RNA isolated during EB differentiation revealed that relative expression levels of the pluripotency factors Nanog and Oct4 were higher in null compared with wild-type EBs over the 6-day period of EB differentiation (Fig. 5A). This was further confirmed by a longer time course experiment of EB differentiation with two independent null ESC lines (Supporting Information Fig. S5B, S5C). In addition, we found that the derepressed primitive endoderm marker *Gata6* in null ESCs (day 0) remained high at most time points of EB differentiation (Supporting Information Fig. S5B, S5C). It is also noteworthy that the trophectoderm marker *Cdx2* was not induced in Zfp281 null EBs throughout the time course of differentiation (Supporting Information Fig. S5B, S5C). Together with the downregulation of additional trophectoderm markers *Elf5* and *Rhox6* and derepression of definitive endoderm markers *Cxcr4* and *Sox17* upon Zfp281 depletion (Supporting Information Fig. S4E), our data suggest that Zfp281 null ESCs may be skewed toward endoderm differentiation and defective in trophectoderm differentiation during EB culture.

### Zfp281 Is Required for Nanog Binding to the *Nanog* Promoter in Transcriptional Regulation

We have shown that Zfp281 mainly acts as a repressor to repress stem cell specific genes (e.g., *Nanog*) for pluripotency maintenance. Zfp281 was copurified with Nanog protein complexes [9] and found to interact with Nanog via its C-terminal domain [13]. The upregulation of Nanog in Zfp281 null ESCs at both the RNA and protein levels (Fig. 2; Supporting Information Fig. S2) prompted us to explore the potential contributions of Zfp281 in transcriptional regulation of *Nanog* in ESCs. Specifically, as the *Nanog* gene is a direct target of its own gene product [4, 5], as well as of Zfp281 [13], we asked if Zfp281, as a partner of Nanog, is required for Nanog binding to its own promoter for transcriptional regulation.

**Figure 6:**
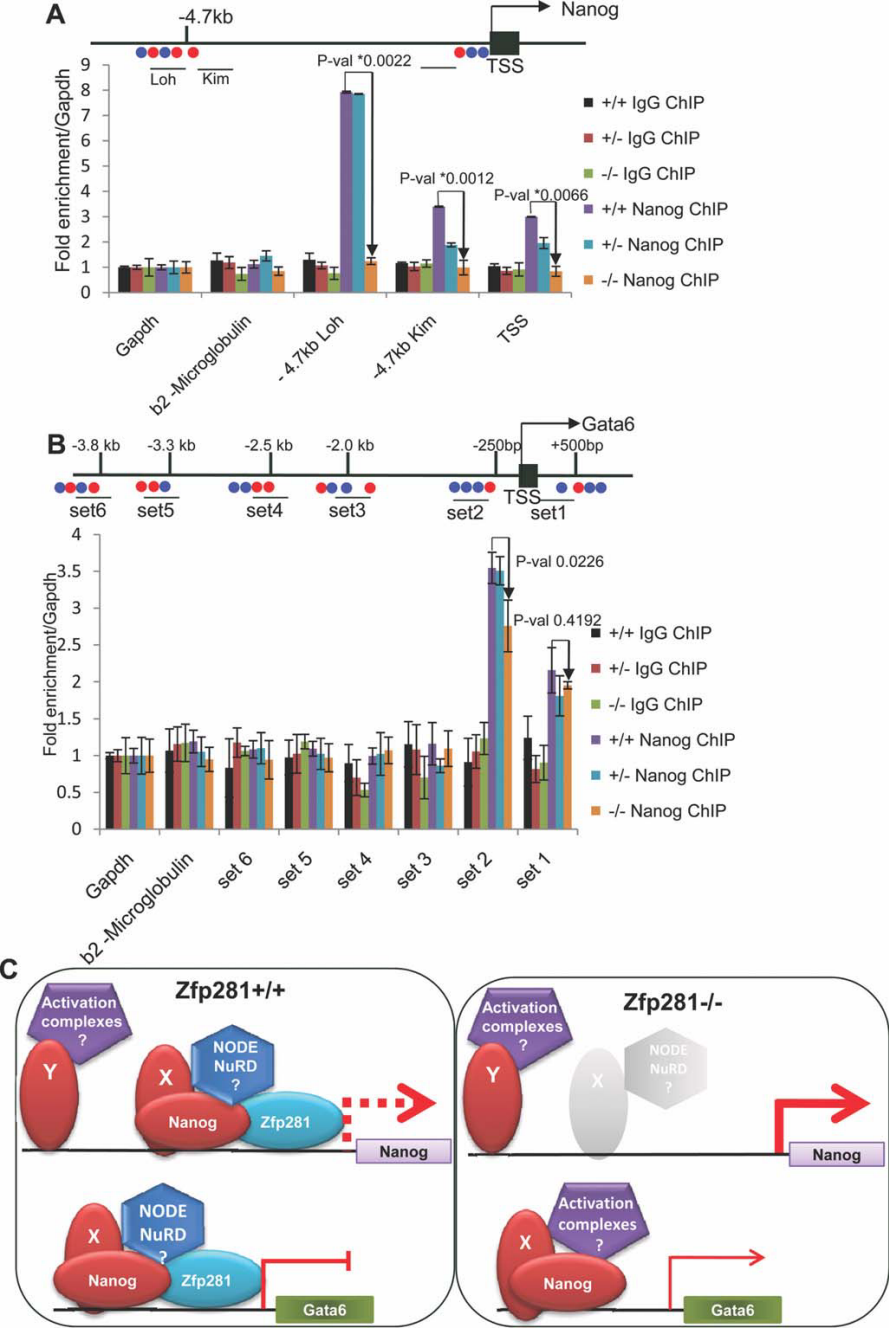
Cooperative binding of Nanog and Zfp281 at the *Nanog*(A) and *Gata6*(B) regulatory regions. (A, B): Chromatin immunoprecipitation (ChIP) experiments were performed using total rabbit IgG or antibody against Nanog in wild-type, heterozygous, and null embryonic stem cells (ESCs). ChIP data were quantified by quantitative polymerase chain reaction (PCR) and normalized to GAPDH using the indicated primer sets (Supporting Information Table S2) for *Nanog*(A) and *Gata6*(B) regulatory regions. β2-microglobulin was used as a negative control. The transcriptional start site was designated as 0. Error bars indicate SD. Blue dots denote putative Zfp281 binding sites and red dots denote putative Nanog binding sites. Short lines underneath the color dots indicate PCR amplicons. *, *p* with statistical significance. (C): A putative model of Zfp281 function in regulating *Nanog* and *Gata6* expression in mouse ESCs. In wild-type ESCs, Zfp281 may be required by Nanog for binding to the regulatory regions of *Nanog* and *Gata6* and to recruit NuRD/NODE repressor complexes. The repressor function of Zfp281 ensures optimal expression of Nanog (indicated by the dashed arrow) and suppresses endoderm differentiation of ESCs. In the absence of Zfp281, Nanog and the repressive complexes are disengaged from the *Nanog* promoter/enhancer region, or Nanog switches its partners from repressive complexes to activating complexes in the *Gata6* promoter, which results in upregulation of *Nanog* expression and derepression of *Gata6* in mutant ESCs.

Prior studies have documented two principal Nanog binding sites within the promoter/enhancer region of the *Nanog* gene: one near the transcription start site (TSS) and the other in a −4.7-kb enhancer region (Fig. 6A, top). Multiple consensus binding sites for Nanog (red dots) and Zfp281 (blue dots) are present in these regions (Fig. 6A, top). Consistent with previous ChIP studies [4, 5], we confirmed the binding of Nanog to both regions by ChIP-PCR in both wild-type and heterozygous Zfp281 ESCs (Fig. 6A, bottom: purple and blue bars). More importantly, in the absence of Zfp281, Nanog binding to both regions is abolished (Fig. 6A, orange bars). These results suggest that binding of Nanog to these regulatory regions of the *Nanog* gene is dependent on Zfp281.

We [9] have previously shown that both Nanog and Zfp281 bind to Intron 1 (approximately 500 bp) of the *Gata6* gene using ^bio^ChIP-PCR [9, 31]. However, using Nanog antibody-based ChIP-PCR, the enrichment of Nanog in this region is merely twofold using the primer pair set one (Fig. 6A), and Nanog binding is unaffected by the loss of Zfp281 (Fig. 6A). Others [32] have also shown by ChIP-PCR that Nanog has the highest enrichment in the −250 bp region of the *Gata6* proximal promoter, where a single Nanog consensus site is preceded by three Zfp281 binding sites (Fig. 6A, top). While we observed a relatively higher enrichment of Nanog in this region (primer pair set two) for both wild-type and heterozygous ESCs compared with the null ESCs, we only detected an insignificant reduction of Nanog binding upon Zfp281 deletion (Fig. 6A, bottom). Our data suggest that Nanog binding to the *Gata6* promoter may not be as strictly dependent on Zfp281 as the *Nanog* enhancer/promoter. Alternatively, Zfp281 may have Nanog-independent repressor function in regulating *Gata6* gene expression (see more in Discussion section).

## DISCUSSION

Despite recent efforts in delineating the Nanog and Oct4 interactomes [7–9], the specific roles of additional factors that interact with Nanog/Oct4 remains to be defined. In this study, we have performed a detailed functional characterization of one of the Nanog interacting proteins, the Krüppel-like zinc finger protein 281 (Zfp281) for its roles in self-renewal and pluripotency of ESCs. Our results show that while Zfp281 is dispensable for establishment and maintenance of ESCs, it is essential for proper differentiation and pluripotency of ESCs. We also showed that Zfp281 mainly functions as a transcriptional repressor of stem cell active genes. In particular, we demonstrated that Zfp281 is required for Nanog binding to its own promoter and thus may provide a mechanism for fine-tuning Nanog expression in maintaining pluripotency.

Previous studies by us [9] and others [13] showed that the stable knockdown of Zfp281 by short hairpin RNA (shRNA) leads to derepression of the primitive endoderm markers *Gata6/4* and compromised proliferation [9] and/or differentiation of ESCs [13]. In particular, our own knockdown study [9] showed derepression of both stem cell markers (*Nanog*, *Oct4*, and *Rex1*) and endodermal markers (*Gata6/4*), consistent with the marker gene changes in our knockout ESCs reported in this study. However, the RNAi study by others [13] demonstrated, to our surprise, that Zfp281 functions as an activator of Nanog, where knockdown of Zfp281 led to downregulation of several stem cell factors including Nanog, Oct4, Sall4, and Esrrb [13]. The reason for such discrepancies is unknown. It may be attributed to the dosage sensitivity of Zfp281 resulting from different knockdown levels in stem cell function and/or off-target effects of the shRNAs. Along this line, it is interesting to note that while Nanog knockout ESCs [28] and knockdown ESCs from one study [33] were reported to maintain an undifferentiated state without loss of pluripotency, Nanog knockdown ESCs from other RNAi studies [34, 35] revealed a differentiation phenotype. As a close partner of Nanog, Zfp281 may have a similar dosage effect as Nanog for ESC maintenance. Nevertheless, detailed marker gene expression from our previous RNAi study [9] and our current study using genetically ablated null alleles argues strongly for a repressive role of Zfp281 in transcriptional regulation of stem cell pluripotency genes. Therefore, our current study provides additional insights into the function of Zfp281 in stem cell control, that is, it is not essential for ESC maintenance but rather it is required for fine-tuning pluripotent gene expression to ensure proper differentiation and thus execution of pluripotency of ESCs.

Many key pluripotency associated factors (e.g., Nanog, Oct4, Sox2, Esrrb, Sall4, and Tcf3) autoregulate their own expression [36], and by doing so, they directly downregulate their own transcription to prevent over activation and hence maintain homeostasis of ESCs. For example, overexpression of Oct4, Sox2, and Tcf3 triggers differentiation, whereas overexpression of Nanog blocks ESC differentiation. However, the mechanism for such autoregulation is less well-defined. Our study demonstrates that Zfp281 principally functions as a repressor rather than as an activator in maintaining stem cell pluripotency, which is consistent with our earlier genome-wide ChIP-on-chip studies in ESCs that showed predicted targets of Zfp281 to be considerably enriched for the repressive H3K27me3 mark [5]. Our current study suggests that Zfp281 may fine-tune optimal expression levels of pluripotency factors in ESCs and keep lineage specific factors (e.g., Gata6) in check by repression.

Of particular interest is the derepression of both *Nanog* and *Gata6* in Zfp281 null ESCs. *Gata6* is also a direct target of Nanog [4, 5] and Zfp281 [9] by ChIP and conventional gel shift assays [4, 26, 32, 37, 38]. Derepression of both *Nanog* and *Gata6* upon Zfp281 depletion is notable as well as paradoxical. It has been shown that the Nanog interactome connects with multiple corepressor pathways [9] including the Mi-2/NuRD repressor complex and another related NODE repressive complex in ESCs [9, 11]. It is tempting to hypothesize that the NuRD/NODE repressive complexes are recruited to the *Nanog* and *Gata6* promoters by Zfp281 to fine-tune Nanog expression and to repress Gata6 expression, respectively, in maintaining optimal self-renewal of ESCs (Fig. 6A, left), and such repression is removed in the absence of Zfp281 leading to upregulation of Nanog and derepression of Gata6 in the null ESCs (Fig. 6A, right). Future studies are warranted to test this hypothesis to unravel the autorepression regulatory mechanism of Nanog for pluripotency of ESCs.

Our study and studies of Zfp281 in other cellular contexts have thus far documented its function principally as a transcriptional repressor [15, 16]. However, we cannot exclude the activator function of Zfp281 in different cellular contexts and throughout various developmental stages, in particular, the trophectoderm lineage specification and trophoblast stem cell development. Nevertheless, the major repressor function of Zfp281 defined in this study is in line with the importance of transcriptional repression for stem cell pluripotency [10], and highlights that a balanced expression of stem cell factors, in addition to the repression of lineage specific factors, is critical for self-renewal and pluripotency of ESCs.

## SUMMARY

We have generated a targeted null allele of Zfp281 and characterized the function of Zfp281 in regulation of self-renewal and pluripotency of ESCs. Contrary to an earlier RNAi study by others that suggested its function in activating the stem cell pluripotency factors Nanog, Oct4, and Sox2, our current data using genetically ablated Zfp281 null ESCs, combined with our previously reported knockdown studies, unambiguously argue for a repressor function of Zfp281 in regulation of major stem cell factors, including Nanog. It is still formally possible that Zfp281 can both positively and negatively regulate expression of other target genes to maintain pluripotency as well as to execute proper differentiation of ESCs. Our data clearly define a critical role of Zfp281 in maintaining pluripotency by functioning as a repressor to prevent excessive expression of the key stem cell factor Nanog, while simultaneously repressing lineage specific gene (e.g., *Gata6*) expression in ESCs.

## Notes

Fig. S1. Derivation and characterization of Zfp281 deficient ESC lines. (A) Southern blot analysis of ESCs lines derived from blastocysts outgrowth using the 5′Probe (see Fig. 1A for detail). The 7.8 kb band corresponds to the null allele with the floxed Neo cassette removed (1lox). (B) Confirmation of the null status of Zfp281 gene expression by RT-PCR. Primers used to detect the transcript are described in Supplementary Table 1. (C) Cell cycle profile of Zfp281 wild-type, heterozygous and null ESCs. (D) Analysis of apoptosis by flow cytometry. Cells were stained with Annexin-V and PI. The proportion of Annexin-V positive and PI negative cells in Zfp281 deficient ESCs remains as low as that of wild-type and heterozygous ESCs. (E) Morphology of representative Zfp281 null ESC colonies. ESCs were grown in standard culture condition and colonies were stained for alkaline phosphatase activity. Note that Zfp281 deficient ESCs are positively stained for AP activity. (F) Growth curve MTT analyses for wild-type, heterozygous and null ESC lines. (G) Colony formation assay of wild-type, heterozygous and null ESCs. Cells were cultured at clonal density in the presence or absence of LIF for six days and colonies were scored as fully undifferentiated (red), mixed or partially differentiated (pink) and fully differentiated (gray) based on the extent of alkaline phosphatase staining. Error bars represent st.dev.. The significant increase of partially differentiated colonies was observed in null ESCs (indicated by p values).

Fig. S2. Dysregulation of pluripotency and lineage specific markers in Zfp281 null ESCs. (A) Expression levels of Zfp281 transcript in wild-type, heterozygous and null ESCs. (B) Western blot analysis showing a relatively higher level of Nanog but a relatively smaller increase of Oct4 expression in Zfp281 null ESC lines. β-Actin was used as a loading control. Western data were scanned and density of target bands was quantified using the ImageJ Software of the National Institutes of Health, USA. Band density was normalized to that of the β-actin loading control. (C) Quantitative RT-PCR for relative expression levels of pluripotency markers in ESCs. (D) Quantitative RT-PCR for relative expression levels of endodermal markers in ESCs. (E) Quantitative RT-PCR for relative expression levels of differentiation markers in ESCs. Error bars represent st.dev.. (F) Independent immunostaining for heterogeneous expression of Nanog in heterozygous, null, and rescued ESCs. Note that expression of Nanog (stained green) in Zfp281 heterozygous and null ESCs is as heterogeneous as that in wild-type ESCs. Three different views for each line are presented with white arrows indicating cells with low or no Nanog expression.

Fig. S3. Gene ontology (GO) analysis of differentially expressed target genes of Zfp281. GO terms for molecular function and biological process are presented for downregulated (A) and upregulated (B) target genes, respectively. The p value for each category is indicated on the right.

Fig. S4. Quantitative PCR validation of differential gene expression in Zfp281 null ESCs. (A-D) Quantitative PCR analyses were performed on two matched pairs of wild-type (WT) and null ESCs (7Null/3WT and 3.38Null/3.3WT) derived from the same litters of early embryos. Data presented in (A) and (C) and in (B) and (D) are from two independent PCR experiments. Downregulated and upregulated genes are indicated with blue and red bars, respectively, for clarity. (E) Quantitative PCR analyses of additional trophectoderm and definitive endoderm markers in two independently derived null ESC lines. Error bars represent st.dev.

Fig. S5. Abnormal in vitro differentiation of Zfp281 deficient ESCs. (A) Morphology (left) and size (right) comparison of embryoid bodies during time course differentiation of wild-type and null ESCs. (B) Quantitative RT-PCR for relative expression levels of pluripotency and differentiation markers during the time course of EB differentiation. (C) Quantitative RT-PCR for relative expression levels of pluripotency and differentiation markers during the time course of EB differentiation of another independent null ESC line. For each gene, expression levels were normalized to wild-type mRNA levels at day 0.
