# Supplementary figures and images for: Zfp281 Functions as a Transcriptional Repressor for Pluripotency of Mouse Embryonic Stem Cells
Source: Stem Cells. 2011 Sep 13;29(11):1705–16. doi: 10.1002/stem.736 (PMC3272666; doi:10.1002/stem.736)

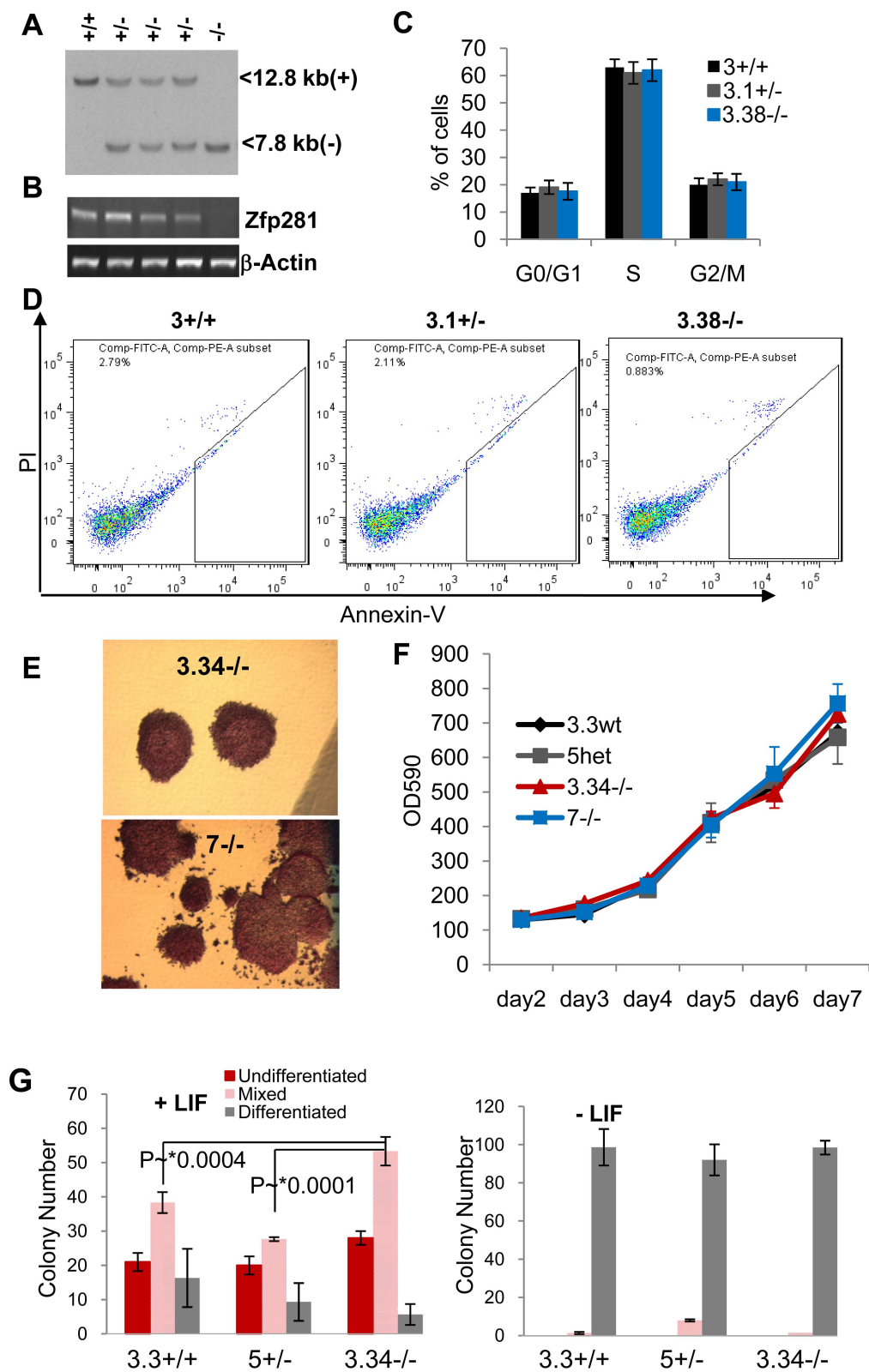

**FIGURE S1**  
Jianlong Wang

Supplement: Supplementary file 1 — Fig. S1. Derivation and characterization of Zfp281 deficient ESC lines. (A) Southern blot analysis of ESCs lines derived from blastocysts outgrowth using the 5′Probe (see Fig. 1A for detail). The 7.8 kb band corresponds to the null allele with the floxed Neo cassette removed (1lox). (B) Confirmation of the null status of Zfp281 gene expression by RT-PCR. Primers used to detect the transcript are described in Supplementary Table 1. (C) Cell cycle profile of Zfp281 wild-type, heterozygous and null ESCs. (D) Analysis of apoptosis by flow cytometry. Cells were stained with Annexin-V and PI. The proportion of Annexin-V positive and PI negative cells in Zfp281 deficient ESCs remains as low as that of wild-type and heterozygous ESCs. (E) Morphology of representative Zfp281 null ESC colonies. ESCs were grown in standard culture condition and colonies were stained for alkaline phosphatase activity. Note that Zfp281 deficient ESCs are positively stained for AP activity. (F) Growth curve MTT analyses for wild-type, heterozygous and null ESC lines. (G) Colony formation assay of wild-type, heterozygous and null ESCs. Cells were cultured at clonal density in the presence or absence of LIF for six days and colonies were scored as fully undifferentiated (red), mixed or partially differentiated (pink) and fully differentiated (gray) based on the extent of alkaline phosphatase staining. Error bars represent st.dev.. The significant increase of partially differentiated colonies was observed in null ESCs (indicated by p values). [file stem0029-1705-SD1.pdf]

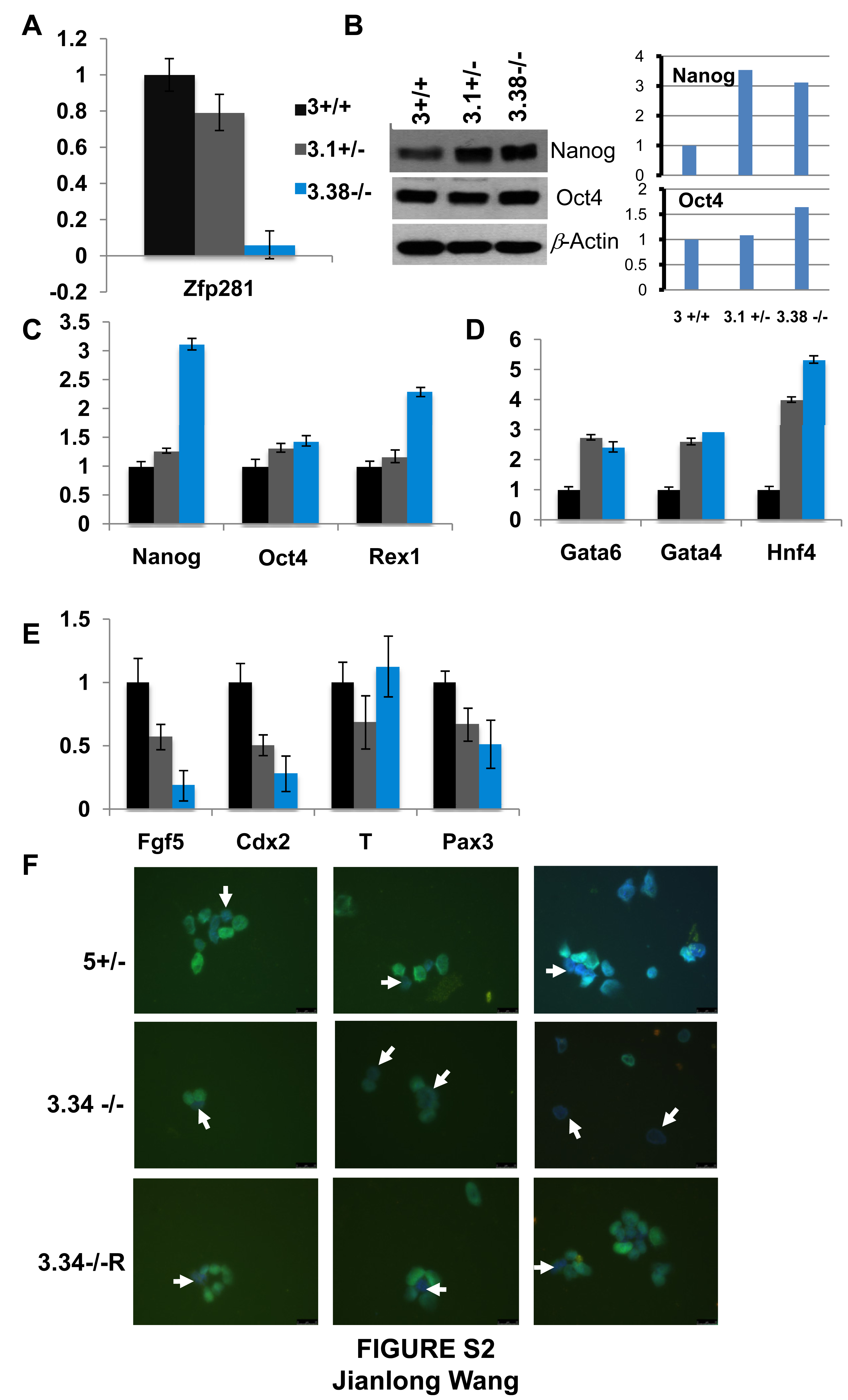

Supplement: Supplementary file 2 — Fig. S2. Dysregulation of pluripotency and lineage specific markers in Zfp281 null ESCs. (A) Expression levels of Zfp281 transcript in wild-type, heterozygous and null ESCs. (B) Western blot analysis showing a relatively higher level of Nanog but a relatively smaller increase of Oct4 expression in Zfp281 null ESC lines. β-Actin was used as a loading control. Western data were scanned and density of target bands was quantified using the ImageJ Software of the National Institutes of Health, USA. Band density was normalized to that of the β-actin loading control. (C) Quantitative RT-PCR for relative expression levels of pluripotency markers in ESCs. (D) Quantitative RT-PCR for relative expression levels of endodermal markers in ESCs. (E) Quantitative RT-PCR for relative expression levels of differentiation markers in ESCs. Error bars represent st.dev.. (F) Independent immunostaining for heterogeneous expression of Nanog in heterozygous, null, and rescued ESCs. Note that expression of Nanog (stained green) in Zfp281 heterozygous and null ESCs is as heterogeneous as that in wild-type ESCs. Three different views for each line are presented with white arrows indicating cells with low or no Nanog expression. [file stem0029-1705-SD2.tif]

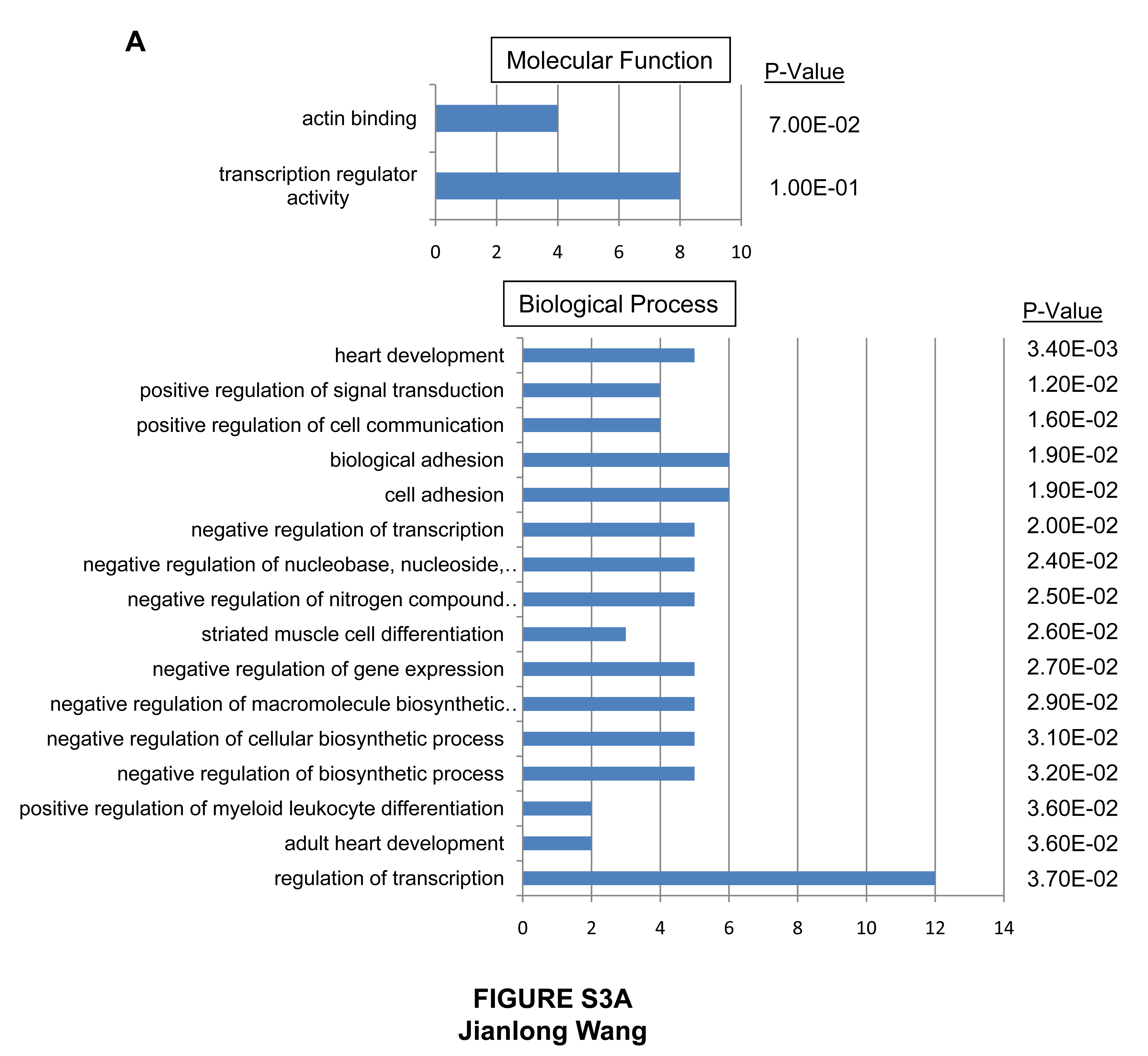

Supplement: Supplementary file 3 — Fig. S3. Gene ontology (GO) analysis of differentially expressed target genes of Zfp281. GO terms for molecular function and biological process are presented for downregulated (A) and upregulated (B) target genes, respectively. The p value for each category is indicated on the right. [file stem0029-1705-SD3A.tif]

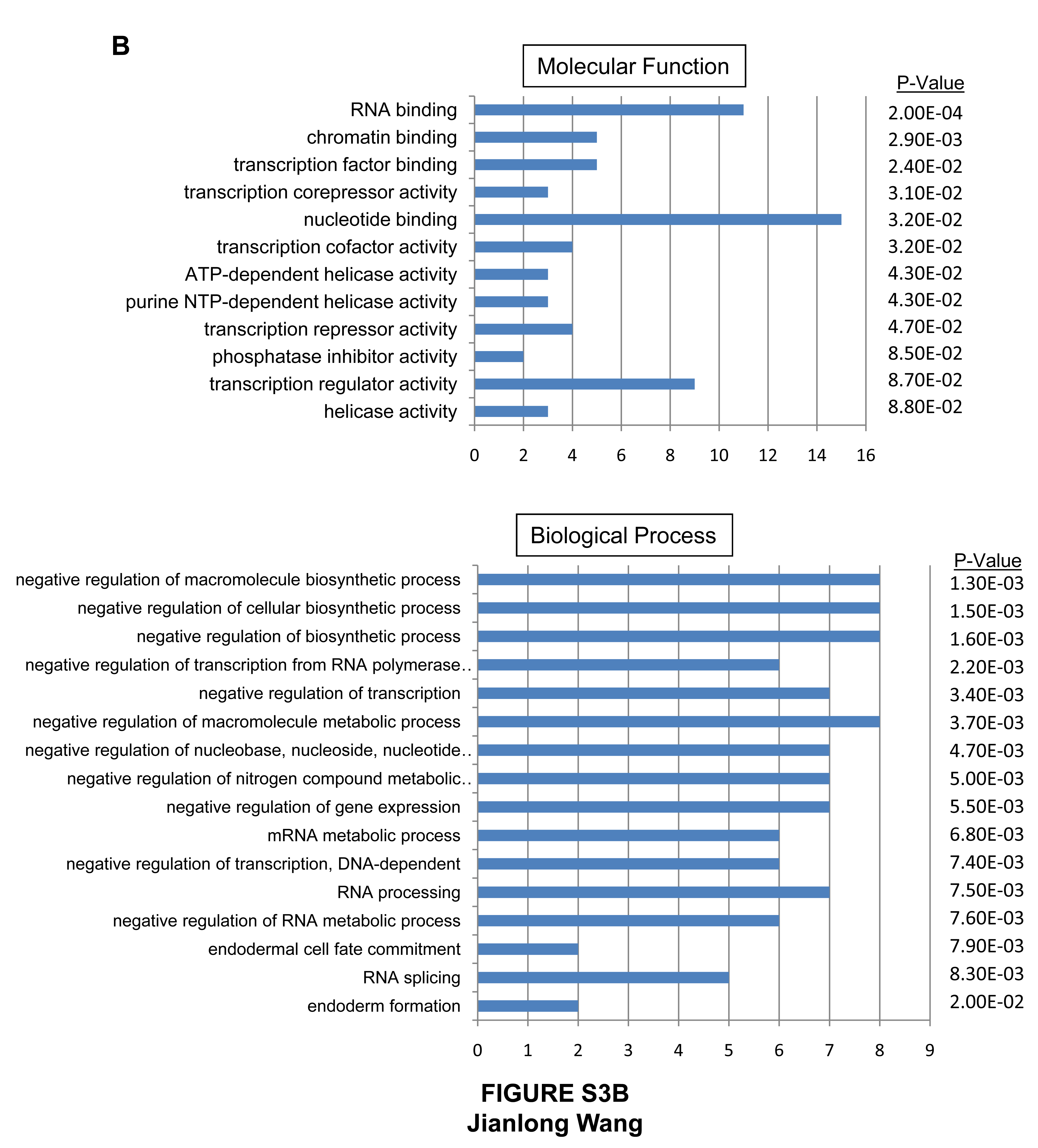

Supplement: Supplementary file 4 — Fig. S3. Gene ontology (GO) analysis of differentially expressed target genes of Zfp281. GO terms for molecular function and biological process are presented for downregulated (A) and upregulated (B) target genes, respectively. The p value for each category is indicated on the right. [file stem0029-1705-SD3B.tif]

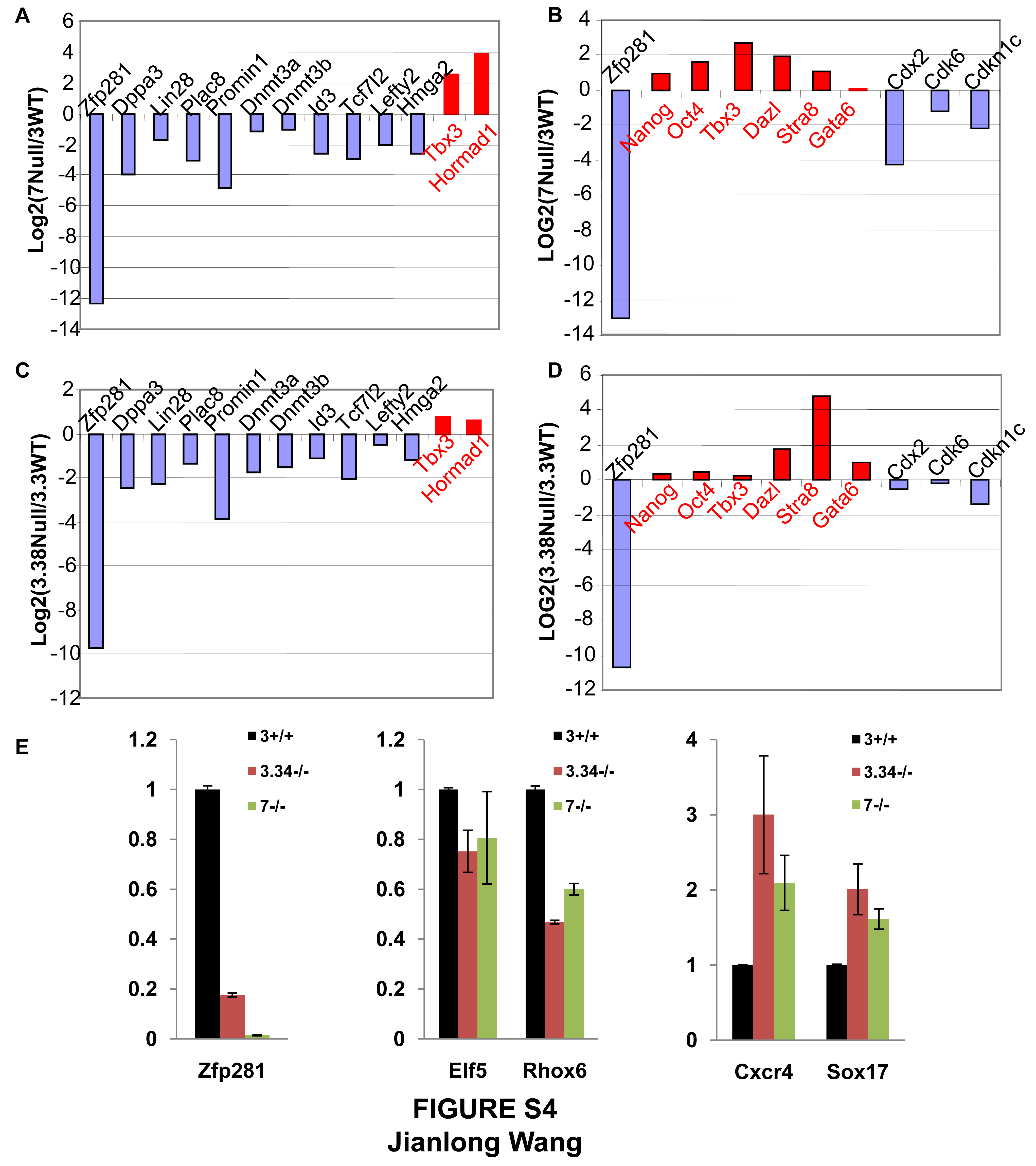

Supplement: Supplementary file 5 — Fig. S4. Quantitative PCR validation of differential gene expression in Zfp281 null ESCs. (A-D) Quantitative PCR analyses were performed on two matched pairs of wild-type (WT) and null ESCs (7Null/3WT and 3.38Null/3.3WT) derived from the same litters of early embryos. Data presented in (A) and (C) and in (B) and (D) are from two independent PCR experiments. Downregulated and upregulated genes are indicated with blue and red bars, respectively, for clarity. (E) Quantitative PCR analyses of additional trophectoderm and definitive endoderm markers in two independently derived null ESC lines. Error bars represent st.dev. [file stem0029-1705-SD4.tif]

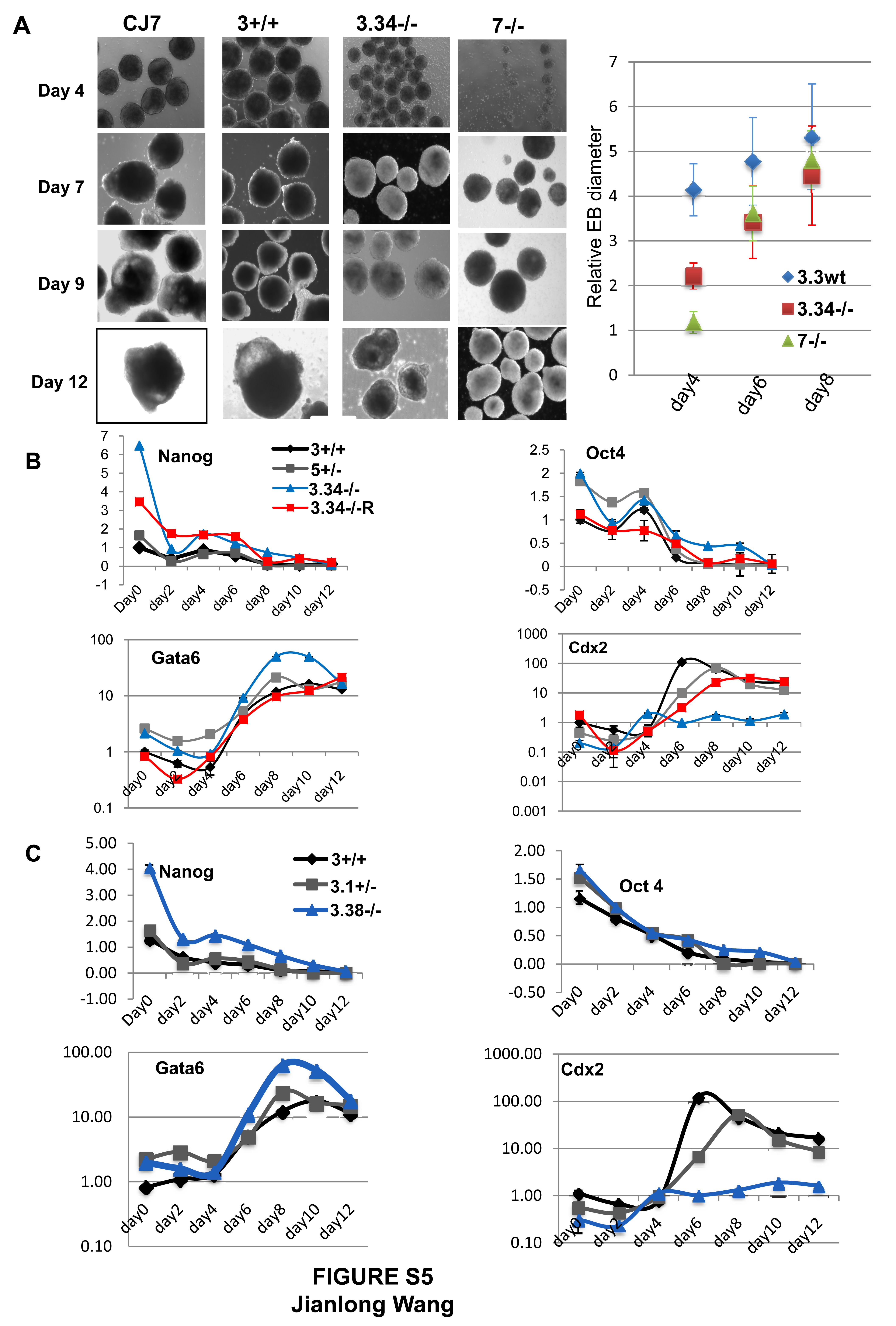

Supplement: Supplementary file 6 — Fig. S5. Abnormal in vitro differentiation of Zfp281 deficient ESCs. (A) Morphology (left) and size (right) comparison of embryoid bodies during time course differentiation of wild-type and null ESCs. (B) Quantitative RT-PCR for relative expression levels of pluripotency and differentiation markers during the time course of EB differentiation. (C) Quantitative RT-PCR for relative expression levels of pluripotency and differentiation markers during the time course of EB differentiation of another independent null ESC line. For each gene, expression levels were normalized to wild-type mRNA levels at day 0. [file stem0029-1705-SD5.tif]

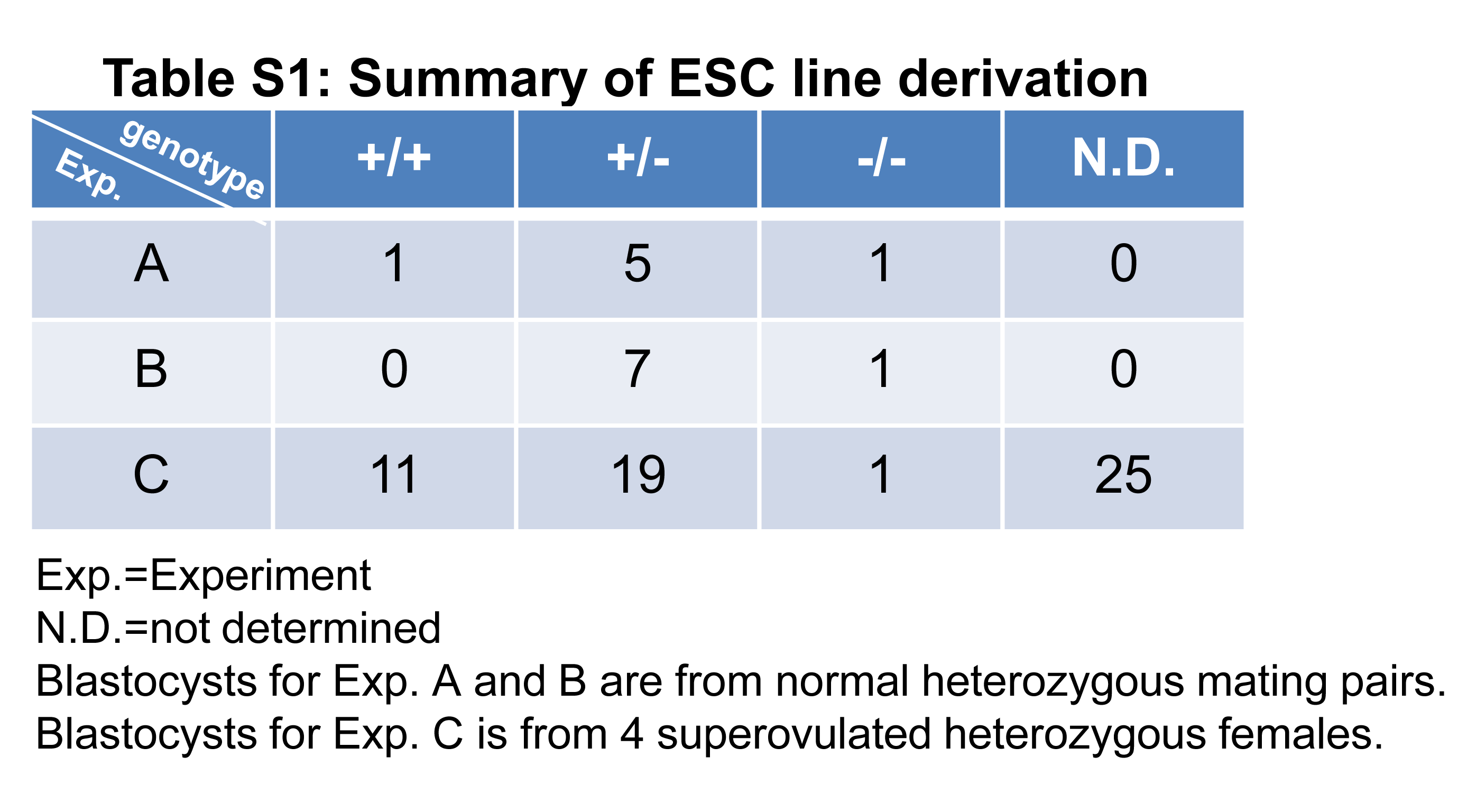

Supplement: Supplementary file 7 [file stem0029-1705-SD6A.tif]
