# Supplementary material for: Zfp281 Functions as a Transcriptional Repressor for Pluripotency of Mouse Embryonic Stem Cells
Source: Stem Cells. 2011 Sep 13;29(11):1705–16. doi: 10.1002/stem.736 (PMC3272666; doi:10.1002/stem.736)
Supplement: Supplementary file 8 [file stem0029-1705-SD6B.pdf]

Supplemental Table S2. Primers Used in This Study

Gene for RT-PCR	Forward primer	Reverse primer	
Zfp281	TGAGCCCAGGCACCCA	TGGAGAGGTGAAGACAAGCTGAC	
Oct4	CTGAGGGCCAGGCAGGAGCACGAG	CTGTAGGGAGGGCTTCGGGCACTT	
Nanog	AGGGTCTGCTACTGAGATGCTCTG	CAACCACTGGTTTTTCTGCCACCG	
Rex1	ACGAGTGGCAGTTTCTTCTTGGGA	TATGACTCACTTCCAGGGGGCACT	
Tbx3	TCTCCATCGTGGGGACAT	TTGTCGCGGCCTGGCTCCTCG	
Gata6	TTGCTCCGGTAACAGCAGTG	GTGGTCGCTTGTGTAGAAGGA	
Gata4	CTGTCATCTCACTATGGGCA	CCAAGTCCGAGCAGGAATTT	
Hnf4a	TCTGGATGACCAGGTGGCGCT	TGCGGATGGACACACGGCTC	
Fgf5	CTCAGGGGATTGTAGGAATACGAGGA	GGATCGCGGACGCATAGGTATTATAG	
Dppa3	GCATTGCAGCCGTACCTGTG	TGGCTCACTGTCCCGTTCAA	
Lin28	CGAAGCCTCAAGGAGGGTGA	TGCATTCCTTGGCATGATGG	
Plac8	ATTCGTTCGTGCTCCCCAAA	GCCACCGTTGTTCCACACAG	
Promin1	AAGATGCAGCCACCCAGCTC	CCCCCTTTTGACGAGGCTCT	
Dnmt3a	ACTTGGAGAAGCGGAGTGAA	CTGTTCTTTGCCCTCTCCTG	
Dnmt3b	ACTTGGTGATTGGTGGAAGC	CCAGAAGAATGGACGGTTGT	
Id3	GCTTGGGACCCTGGGACTCT	AGGGTGGGGACAGAGTGACG	
Tcf7l2	GGCGCTAACGACGAGCTGAT	CTCTTGGCCGCTTCTTCCAA	
Lefty2	GTGGCTTTGCTGGGCACTCT	CCTCACGTGGGTAGGGATGG	
Hmga2	ACCCAGGAAGCAGCAGCAAG	GCGGACTCTTGCGAGGATGT	
Hormad1	CGCTTCCCTCAGGCGAAAAT	TGTCCCATAAGCACGTTCTGGA	
Stra8	TGACTCGTCAGGGCTTCGTG	CTACCAGGGTGGCTCGATGG	
Cdk6	AGCCTGAAGCCAGCATGGAG	TTGGGGTGCTCGAAGGTCTC	
Cdkn1c	TGGACAGGACAAGCGATCCA	GCCCAGCTCCTCGTGGTCTA	
Dazl	TCATGTGCAGCCACGTCCTT	CTGCTCTCCAGCAGGCCACT	
T	GCTTCAAGGAGCTAACTAACGAG	CCAGCAAGAAAGAGTACATGGC	
Pax3	ATAAGCCCAGGACACAGAGTTGTG	GTCTAGTCTGTGGAGGCCGGAAAC	
Cdx2	CCTGCGACAAGGGCTTGTTTAG	TCCCGACTTCCCTTCACCATAC	
Afp	GAGTGCTTCCAGACAAAGAG	ATAATGGTTGTTGCCTGGAG	
Cxcr4	AGGACCTGTGCCAAGTTCTTAGT	CCAGCATTTCTACCACCATTTCAG	
Sox17	CGCACGGAATTCGAACAGTA	GTCAAATGTCGGGGTAGTTG	

Rhox6	GAATTGGTTTCGGATGAGGA	GTGGCTCAGAAGAAGCCATC	
Elf5	CCCTCCTCCTCTTCAAAACC	AAGTTGCCACAAGACCATCC	
Gapdh	ACCCAGAAGACTGTGGATGG	CACATTGGGGGTAGGAACAC	
**ChIP PCR region**	**Forward Primer**	**Reverse Primer**	**Co-ordinates from TSS**
Gapdh	AAGCTCATGAGGCACAGAATGGTC	TGGGTACATGGTGACTTTCCTAGGC	
β2-Microglobulin	TCAGGTCCTAAGTCCTTTTCTGAGTGG	TGGACCCTTGGTGCCCTACTATCTAG	
Nanog TSS	AATGAGGTAAAGCCTCTTTTTGG	ACCATGGACATTGTAATGCAAA	-130bp
Nanog kim	TGGGGTAAACTTAAGGCTATGG	AGCTCTAAGCCGGTTCTCATTT	-4.55kb
Nanog Loh	ACTCCAAGGCTAGCGATTCA	AATAGGGAGGAGGGCGTCTA	-4.86kb
Gata6 Set1	CAGGAGCGCAGGCTGCAGTT	AGAAGAACGGCCGCGACCGA	+92bp
Gata6 Set 2	TGACCCAGGAGGGGGCGAGT	CCGCCACCCAGGGCAGAAGA	-317bp
Gata6 Set 3	CCAGAGGCGCCAGGGTCAGT	GCTCCGACGCGAGCCTAGTT	-2.08kb
Gata6 Set 4	CCGCACCCCGGATTCTGCTG	CGGACTCTGGCGACGACACC	-2.60kb
Gata6 Set 5	AGCGATACACATCCAGGCCCG	GGGCGGGGGCTCTGTAGCTA	-3.05kb
Gata6 Set 6	CGCCCAGCGTGGGGCTTTTA	TGTCCGCGCGCTGTTTACGT	-3.98kb
