# Supplementary material for: Zfp281 Functions as a Transcriptional Repressor for Pluripotency of Mouse Embryonic Stem Cells
Source: Stem Cells. 2011 Sep 13;29(11):1705–16. doi: 10.1002/stem.736 (PMC3272666; doi:10.1002/stem.736)
Supplement: Supplementary file 9 [file stem0029-1705-SD6C.pdf]

Supplemental Table S3. A Complete List of Genes Shown in Fig. 4C

NAME	PROBE	GENE SYMBOL	GENE_TITLE	RANK IN GENE LIST	RANK METRIC SCORE	RUNNING ES	CORE ENRICHMENT
row_0	CHD7	CHD7	chromodomain helicase DNA binding protein 7	121	0.491048157	0.013122713	No
row_1	GLT28D1	GLT28D1	glycosyltransferase 28 domain containing 1	330	0.342615634	0.01659919	No
row_2	DNMT3B	DNMT3B	DNA (cytosine-5-)-methyltransferase 3 beta	463	0.298328817	0.021883564	No
row_3	TCFAP2C	null	null	470	0.296753198	0.032898962	No
row_4	PSIP1	PSIP1	PC4 and SFRS1 interacting protein 1	765	0.244092599	0.028674169	No
row_5	SATB1	SATB1	special AT-rich sequence binding protein 1 (binds to nuclear matrix/scaffold-associating DNA's)	792	0.240699232	0.036637567	No
row_6	SNURF	SNURF	SNRPN upstream reading frame	1206	0.204330549	0.025430612	No
row_7	TRH	TRH	thyrotropin-releasing hormone	1336	0.194972292	0.026920257	No
row_8	ERAS	ERAS	ES cell expressed Ras	1433	0.188039571	0.029662803	No
row_9	SALL4	SALL4	sal-like 4 (Drosophila)	2087	0.155640244	0.005572807	No
row_10	RBPMS2	RBPMS2	RNA binding protein with multiple splicing 2	2952	0.128441319	-0.029249664	No
row_11	ITPR1	ITPR1	inositol 1,4,5-triphosphate receptor, type 1	3395	0.116241693	-0.045141153	No
row_12	ANP32A	ANP32A	acidic (leucine-rich) nuclear phosphoprotein 32 family, member A	3776	0.107845291	-0.05850259	No
row_13	TMEM55B	TMEM55B	transmembrane protein 55B	3797	0.107448027	-0.055333503	No
row_14	SNRPN	SNRPN	small nuclear ribonucleoprotein polypeptide N	6505	0.061767787	-0.17739731	No
row_15	ATF7IP	ATF7IP	activating transcription factor 7 interacting protein	6518	0.061665889	-0.17560251	No
row_16	PDK1	PDK1	pyruvate dehydrogenase kinase, isozyme 1	6714	0.059431296	-0.18230343	No
row_17	MTAP7	null	null	7178	0.053532049	-0.20154612	No
row_18	RPS8	RPS8	ribosomal protein S8	7258	0.05224485	-0.2031891	No
row_19	ZRANB3	ZRANB3	zinc finger, RAN-binding domain containing 3	7305	0.051455405	-0.20334543	No
row_20	IFITM1	IFITM1	interferon induced transmembrane protein 1 (9-27)	7310	0.051400036	-0.20157357	No
row_21	CDCA5	CDCA5	cell division cycle associated 5	7806	0.045140654	-0.22260626	No
row_22	SAP30	SAP30	Sin3A-associated protein, 30kDa	8151	0.040913802	-0.2368598	No
row_23	SOCS2	SOCS2	suppressor of cytokine signaling 2	8217	0.040074095	-0.23832244	No
row_24	SET	SET	SET translocation (myeloid leukemia-associated)	8602	0.035196245	-0.25463194	No

row_25	DDX5	DDX5	DEAD (Asp-Glu-Ala-Asp) box polypeptide 5	8797	0.032882299	-0.26229706	No
row_26	DTNB	DTNB	dystrobrevin, beta	8936	0.030956971	-0.26746166	No
row_27	SUMO3	SUMO3	SMT3 suppressor of mif two 3 homolog 3 (S. cerevisiae)	9237	0.027616994	-0.2801989	No
row_28	HELB	HELB	helicase (DNA) B	9332	0.026628224	-0.28350598	No
row_29	SERBP1	SERBP1	SERPINE1 mRNA binding protein 1	9811	0.021656102	-0.3046509	No
row_30	USP1	USP1	ubiquitin specific peptidase 1	9846	0.021147238	-0.30540892	No
row_31	CDCA2	CDCA2	cell division cycle associated 2	9941	0.020090438	-0.30896476	No
row_32	MAT2A	MAT2A	methionine adenosyltransferase II, alpha	10017	0.019158674	-0.3116828	No
row_33	RTEL1	RTEL1	regulator of telomere elongation helicase 1	10598	0.012642827	-0.33785862	No
row_34	RPL22	RPL22	ribosomal protein L22	11042	0.008221313	-0.35790613	No
row_35	TCEA3	TCEA3	transcription elongation factor A (SII), 3	11272	0.005894694	-0.3682067	No
row_36	NDG2	null	null	11961	-0.001813257	-0.39975825	No
row_37	PRPF3	PRPF3	PRP3 pre-mRNA processing factor 3 homolog (S. cerevisiae)	12218	-0.005089805	-0.4113304	No
row_38	NOLA3	NOLA3	nucleolar protein family A, member 3 (H/ACA small nucleolar RNPs)	13688	-0.022528661	-0.4779886	No
row_39	D11ERTD636E	null	null	14197	-0.029495742	-0.50021404	No
row_40	EIF4A2	EIF4A2	eukaryotic translation initiation factor 4A, isoform 2	14511	-0.033658732	-0.5133189	No
row_41	TNFSF13	TNFSF13	tumor necrosis factor (ligand) superfamily, member 13	15104	-0.042603198	-0.5389063	No
row_42	EIF2S2	EIF2S2	eukaryotic translation initiation factor 2, subunit 2 beta, 38kDa	15158	-0.043515656	-0.53968644	No
row_43	GLI2	GLI2	GLI-Kruppel family member GLI2	15265	-0.044932526	-0.5428485	No
row_44	ADAM23	ADAM23	ADAM metallopeptidase domain 23	15725	-0.05214088	-0.56196034	No
row_45	SOCS3	SOCS3	suppressor of cytokine signaling 3	15780	-0.053134516	-0.5624205	No
row_46	6720458F09RIK	null	null	15837	-0.054061946	-0.5629372	No
row_47	RPL7L1	RPL7L1	ribosomal protein L7-like 1	15948	-0.055758376	-0.5658713	No
row_48	KLF9	KLF9	Kruppel-like factor 9	16311	-0.061642118	-0.5801634	No
row_49	MTF2	MTF2	metal response element binding transcription factor 2	16366	-0.062582664	-0.58026403	No
row_50	SPP1	SPP1	secreted phosphoprotein 1 (osteopontin, bone sialoprotein I, early T-lymphocyte activation 1)	16438	-0.063809246	-0.58109933	No
row_51	UTF1	**UTF1**	undifferentiated embryonic cell transcription factor 1	16462	-0.064121224	-0.5797167	No

row_52	SOX2	**SOX2**	SRY (sex determining region Y)-box 2	16945	-0.072903521	-0.5990955	No
row_53	CEP78	CEP78	centrosomal protein 78kDa	17219	-0.078515314	-0.6086552	No
row_54	PARP2	PARP2	poly (ADP-ribose) polymerase family, member 2	17637	-0.087663405	-0.6244851	No
row_55	PARP1	PARP1	poly (ADP-ribose) polymerase family, member 1	17660	-0.088166259	-0.6221416	No
row_56	MCM5	MCM5	MCM5 minichromosome maintenance deficient 5, cell division cycle 46 (S. cerevisiae)	18225	-0.101746246	-0.64419174	No
row_57	CENPA	CENPA	centromere protein A	18283	-0.103142962	-0.642887	No
row_58	2410137M14RIK	null	null	18324	-0.104371876	-0.64075416	No
row_59	BXDC5	BXDC5	brix domain containing 5	18481	-0.108614817	-0.64379126	No
row_60	NFYB	NFYB	nuclear transcription factor Y, beta	18521	-0.10969843	-0.64140975	No
row_61	IFITM2	IFITM2	interferon induced transmembrane protein 2 (1-8D)	18729	-0.115985543	-0.64651036	Yes
row_62	SFXN1	SFXN1	sideroflexin 1	18768	-0.11710231	-0.6438012	Yes
row_63	2010315L10RIK	null	null	18827	-0.118882425	-0.6419436	Yes
row_64	LRRC28	LRRC28	leucine rich repeat containing 28	18942	-0.122419268	-0.6425251	Yes
row_65	ZSWIM1	ZSWIM1	zinc finger, SWIM-type containing 1	19039	-0.12601079	-0.64214265	Yes
row_66	TRIB3	TRIB3	tribbles homolog 3 (Drosophila)	19075	-0.127131447	-0.63891405	Yes
row_67	NANOG	**NANOG**	Nanog homeobox	19309	-0.137075484	-0.64440715	Yes
row_68	PRMT3	PRMT3	protein arginine methyltransferase 3	19319	-0.137417853	-0.63959223	Yes
row_69	EEF2	EEF2	eukaryotic translation elongation factor 2	19535	-0.146405503	-0.6439031	Yes
row_70	2810410M20RIK	null	null	19585	-0.148272604	-0.6405135	Yes
row_71	D16ERTD472E	null	null	19687	-0.15261592	-0.63934857	Yes
row_72	CDCA7	CDCA7	cell division cycle associated 7	19704	-0.153275639	-0.63425195	Yes
row_73	RBM13	RBM13	RNA binding motif protein 13	19879	-0.161847979	-0.6360909	Yes
row_74	WHSC2	WHSC2	Wolf-Hirschhorn syndrome candidate 2	19893	-0.162809595	-0.6304936	Yes
row_75	INPP5D	INPP5D	inositol polyphosphate-5-phosphatase, 145kDa	20023	-0.171022817	-0.6299152	Yes
row_76	CTBP2	CTBP2	C-terminal binding protein 2	20032	-0.171692967	-0.62375015	Yes
row_77	FBXO15	**FBXO15**	F-box protein 15	20088	-0.175266832	-0.61960924	Yes
row_78	HIRIP3	HIRIP3	HIRA interacting protein 3	20158	-0.180329308	-0.6159191	Yes
row_79	SUV420H2	SUV420H2	suppressor of variegation 4-20 homolog 2 (Drosophila)	20211	-0.183906779	-0.61131155	Yes
row_80	EZH2	EZH2	enhancer of zeste homolog 2 (Drosophila)	20238	-0.186137646	-0.60542417	Yes
row_81	IFITM3	IFITM3	interferon induced transmembrane protein 3 (1-8U)	20256	-0.187499896	-0.5990713	Yes
row_82	BCAT1	BCAT1	branched chain aminotransferase 1, cytosolic	20332	-0.192903802	-0.59517854	Yes

row_83	MSH6	MSH6	mutS homolog 6 (E. coli)	20334	-0.193132266	-0.587876	Yes
row_84	PRPS1	PRPS1	phosphoribosyl pyrophosphate synthetase 1	20552	-0.209698319	-0.5898706	Yes
row_85	MKRN1	MKRN1	makorin, ring finger protein, 1	20574	-0.210847974	-0.58281314	Yes
row_86	RDBP	RDBP	RD RNA binding protein	20576	-0.21099636	-0.5748309	Yes
row_87	ZFP42	**ZFP42**	zinc finger protein 42 homolog (mouse)	20591	-0.212323233	-0.5673957	Yes
row_88	1110001J03RIK	null	null	20615	-0.214697197	-0.5602837	Yes
row_89	1700019D03RIK	null	null	20671	-0.220182672	-0.5544338	Yes
row_90	DNMT3L	DNMT3L	DNA (cytosine-5-)-methyltransferase 3-like	20672	-0.220273256	-0.54605263	Yes
row_91	BAT1A	null	null	20746	-0.228461891	-0.540715	Yes
row_92	PHC1	PHC1	polyhomeotic homolog 1 (Drosophila)	20784	-0.232668951	-0.5335627	Yes
row_93	GSTA4	GSTA4	glutathione S-transferase A4	20828	-0.236806855	-0.5265287	Yes
row_94	TXNIP	TXNIP	thioredoxin interacting protein	20845	-0.238375276	-0.51819414	Yes
row_95	ZNRD1	ZNRD1	zinc ribbon domain containing 1	20873	-0.241134763	-0.5102601	Yes
row_96	5730590G19RIK	null	null	20882	-0.242192239	-0.50141263	Yes
row_97	DPPA4	DPPA4	developmental pluripotency associated 4	20900	-0.244271606	-0.4928997	Yes
row_98	EXOSC2	EXOSC2	exosome component 2	20929	-0.247778371	-0.48475885	Yes
row_99	KLF2	KLF2	Kruppel-like factor 2 (lung)	20973	-0.252423555	-0.47713068	Yes
row_100	SF3B5	SF3B5	splicing factor 3b, subunit 5, 10kDa	21115	-0.271789193	-0.47326973	Yes
row_101	NUDT1	NUDT1	nudix (nucleoside diphosphate linked moiety X)-type motif 1	21135	-0.274910897	-0.4636829	Yes
row_102	SLC7A3	SLC7A3	solute carrier family 7 (cationic amino acid transporter, y+ system), member 3	21175	-0.284072369	-0.45466667	Yes
row_103	1190003J15RIK	null	null	21199	-0.289055347	-0.4447255	Yes
row_104	TCFCP2L1	null	null	21231	-0.295461208	-0.43490827	Yes
row_105	NODAL	NODAL	nodal homolog (mouse)	21259	-0.299845278	-0.42474037	Yes
row_106	RFC5	RFC5	replication factor C (activator 1) 5, 36.5kDa	21293	-0.305045396	-0.41465038	Yes
row_107	SLC7A7	SLC7A7	solute carrier family 7 (cationic amino acid transporter, y+ system), member 7	21309	-0.308559835	-0.4035994	Yes
row_108	ELOVL6	ELOVL6	ELOVL family member 6, elongation of long chain fatty acids (FEN1/Elo2, SUR4/Elo3-like, yeast)	21312	-0.308906019	-0.39193776	Yes
row_109	HEATR1	HEATR1	HEAT repeat containing 1	21316	-0.309744179	-0.38029018	Yes
row_110	ITPK1	ITPK1	inositol 1,3,4-triphosphate 5/6 kinase	21359	-0.32028991	-0.3700338	Yes
row_111	CHAC1	CHAC1	ChaC, cation transport regulator homolog 1 (E. coli)	21361	-0.320557564	-0.3578829	Yes
row_112	EMG1	EMG1	EMG1 nucleolar protein homolog (S. cerevisiae)	21362	-0.320598304	-0.34568444	Yes

row_113	SLC7A5	SLC7A5	solute carrier family 7 (cationic amino acid transporter, y+ system), member 5	21472	-0.355196297	-0.33717924	Yes
row_114	POU5F1	**POU5F1**	POU domain, class 5, transcription factor 1	21481	-0.356226653	-0.32399285	Yes
row_115	2410146L05RIK	null	null	21486	-0.357585788	-0.31057093	Yes
row_116	1810009A15RIK	null	null	21492	-0.358998835	-0.29714116	Yes
row_117	E130014J05RIK	null	null	21555	-0.38197422	-0.28545696	Yes
row_118	SLC29A1	SLC29A1	solute carrier family 29 (nucleoside transporters), member 1	21576	-0.390425265	-0.27152088	Yes
row_119	GDF3	GDF3	growth differentiation factor 3	21611	-0.402611822	-0.25776455	Yes
row_120	LSG1	LSG1	large subunit GTPase 1 homolog (S. cerevisiae)	21636	-0.41734761	-0.24298795	Yes
row_121	PFKP	PFKP	phosphofructokinase, platelet	21649	-0.423967302	-0.22740793	Yes
row_122	TDH	TDH	L-threonine dehydrogenase	21674	-0.436819315	-0.21189044	Yes
row_123	JAM2	JAM2	junctional adhesion molecule 2	21681	-0.441345453	-0.19537346	Yes
row_124	KLHDC4	KLHDC4	kelch domain containing 4	21686	-0.444082767	-0.1786604	Yes
row_125	RPA2	RPA2	replication protein A2, 32kDa	21706	-0.457072049	-0.16214252	Yes
row_126	ALDH2	ALDH2	aldehyde dehydrogenase 2 family (mitochondrial)	21708	-0.459677011	-0.14469823	Yes
row_127	TEX19	null	null	21738	-0.476422101	-0.12790368	Yes
row_128	UPP1	UPP1	uridine phosphorylase 1	21805	-0.550687551	-0.10998396	Yes
row_129	COBL	COBL	cordon-bleu homolog (mouse)	21846	-0.65298897	-0.08697679	Yes
row_130	RUVBL1	RUVBL1	RuvB-like 1 (E. coli)	21855	-0.686460555	-0.06122534	Yes
row_131	VEGFC	VEGFC	vascular endothelial growth factor C	21865	-0.717081189	-0.03435477	Yes
row_132	GBX2	GBX2	gastrulation brain homeobox 2	21888	-0.931904852	9.21E-05	Yes
